# Supplementary figures and images for: Dimerization of kringle 1 domain from hepatocyte growth factor/scatter factor provides a potent MET receptor agonist
Source: Life Sci Alliance. 2022 Jul 29;5(12):e202201424. doi: 10.26508/lsa.202201424 (PMC9348577; doi:10.26508/lsa.202201424)

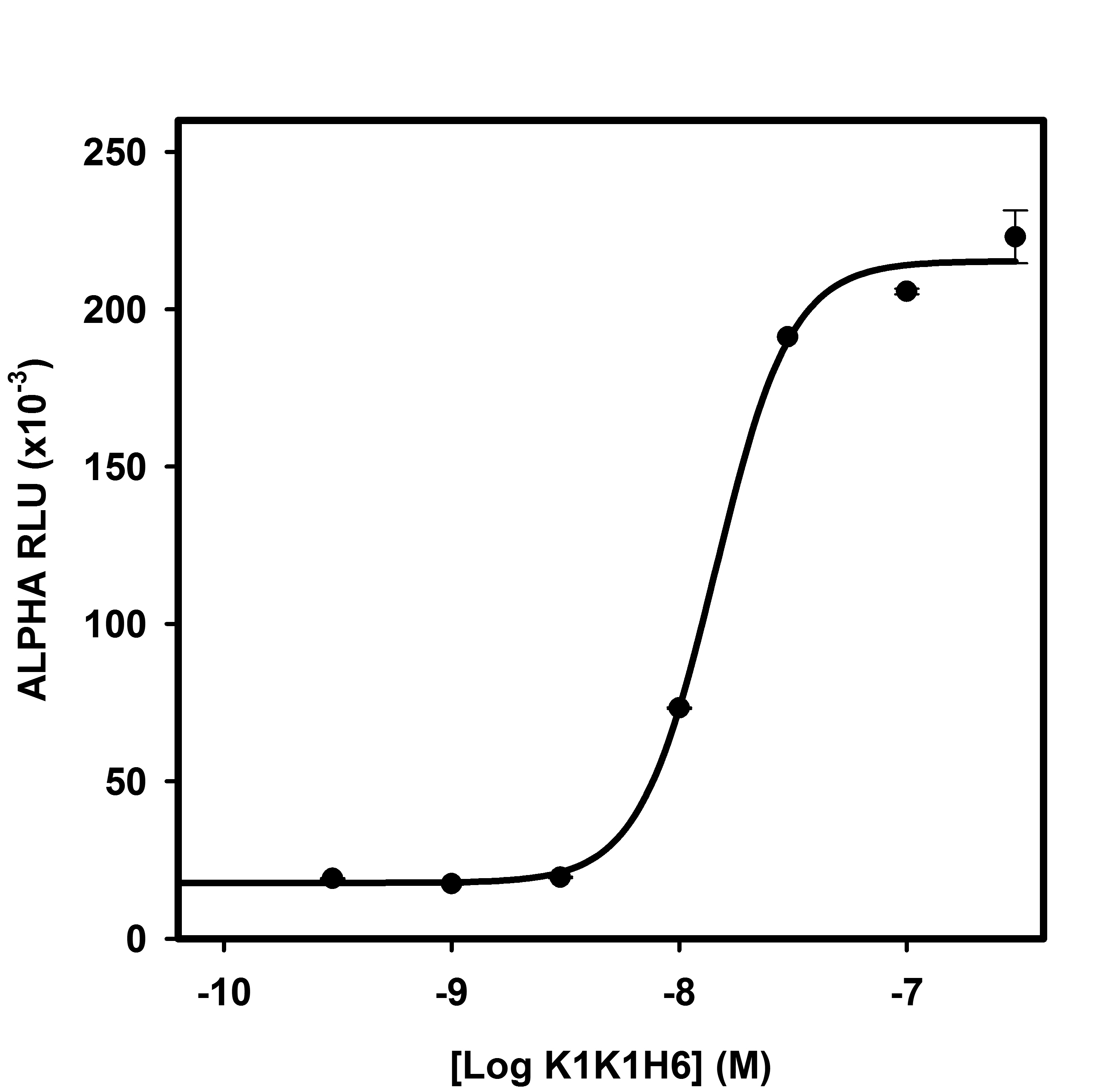

Supplement: Supplementary file 1 [file LSA-2022-01424_SdataF5.3_F6_F7_F8_FS1_FS2_FS3.zip › FigS3A.JPG]

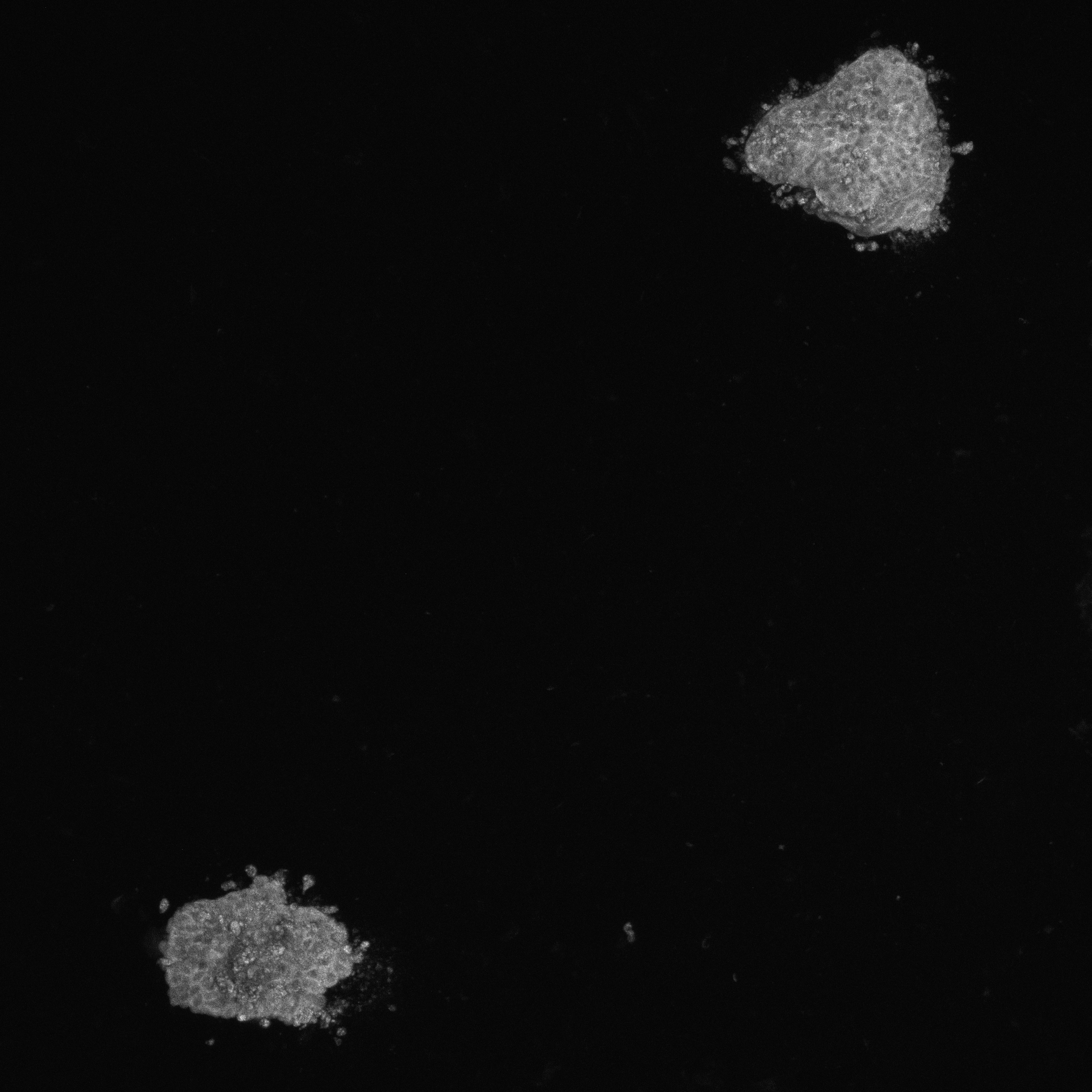

Supplement: Supplementary file 2 [file LSA-2022-01424_SdataF6.2_F7_F8_FS1_FS2_FS3_FS4_FS5.zip › Ctrl x10_Maximum intensity projection.tif]

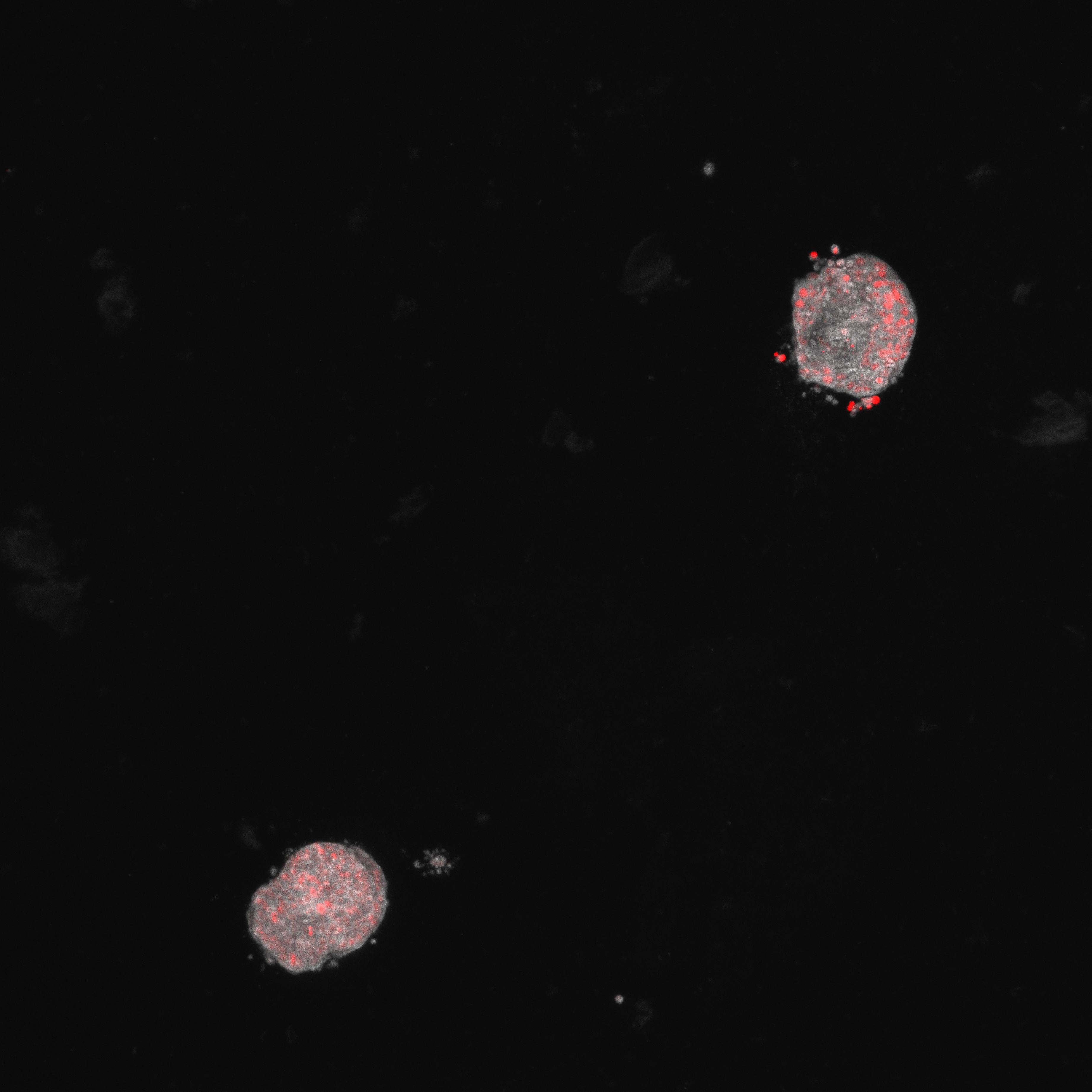

Supplement: Supplementary file 2 [file LSA-2022-01424_SdataF6.2_F7_F8_FS1_FS2_FS3_FS4_FS5.zip › Ctrl_Maximum intensity projection.tif]

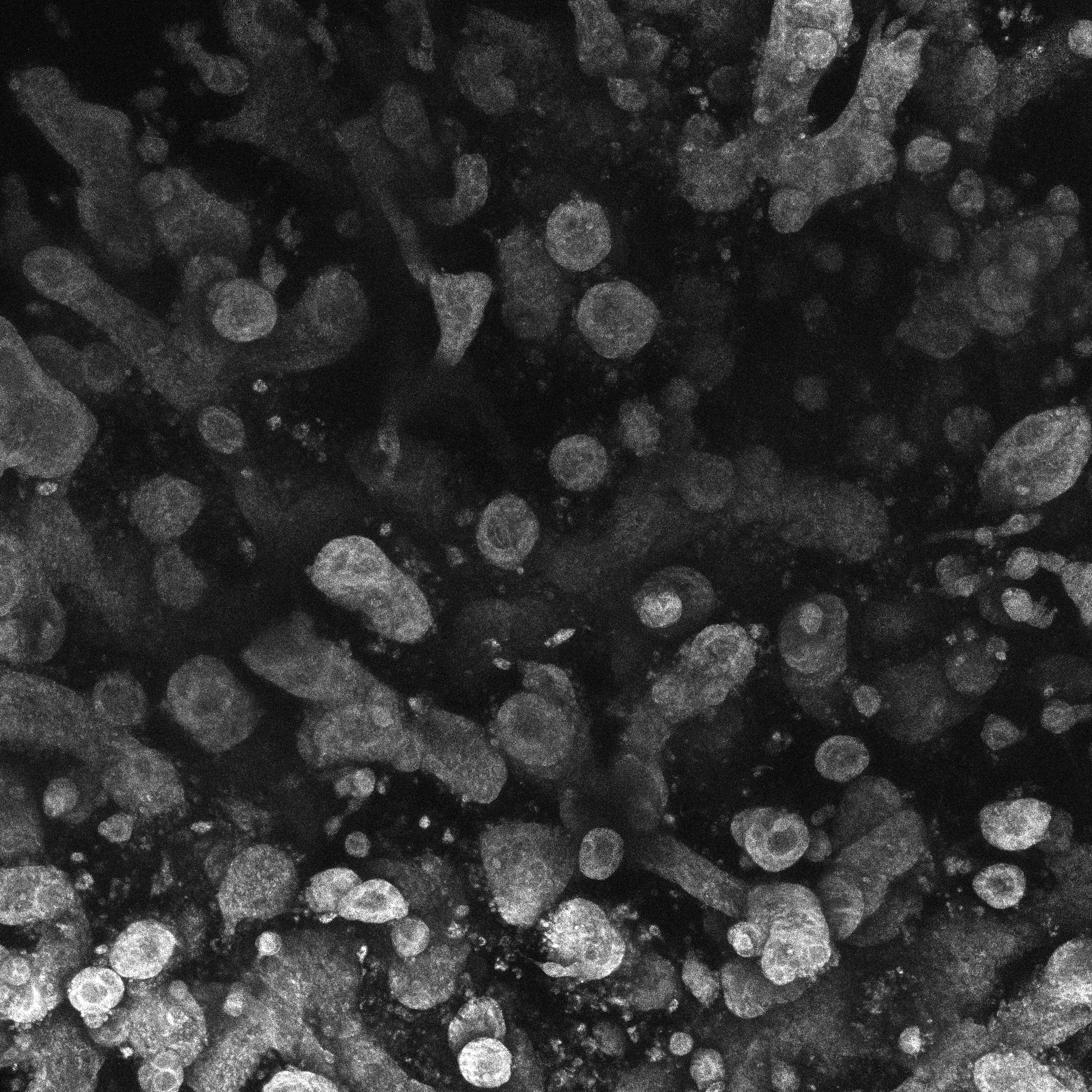

Supplement: Supplementary file 2 [file LSA-2022-01424_SdataF6.2_F7_F8_FS1_FS2_FS3_FS4_FS5.zip › HGF x10_Maximum intensity projection.tif]

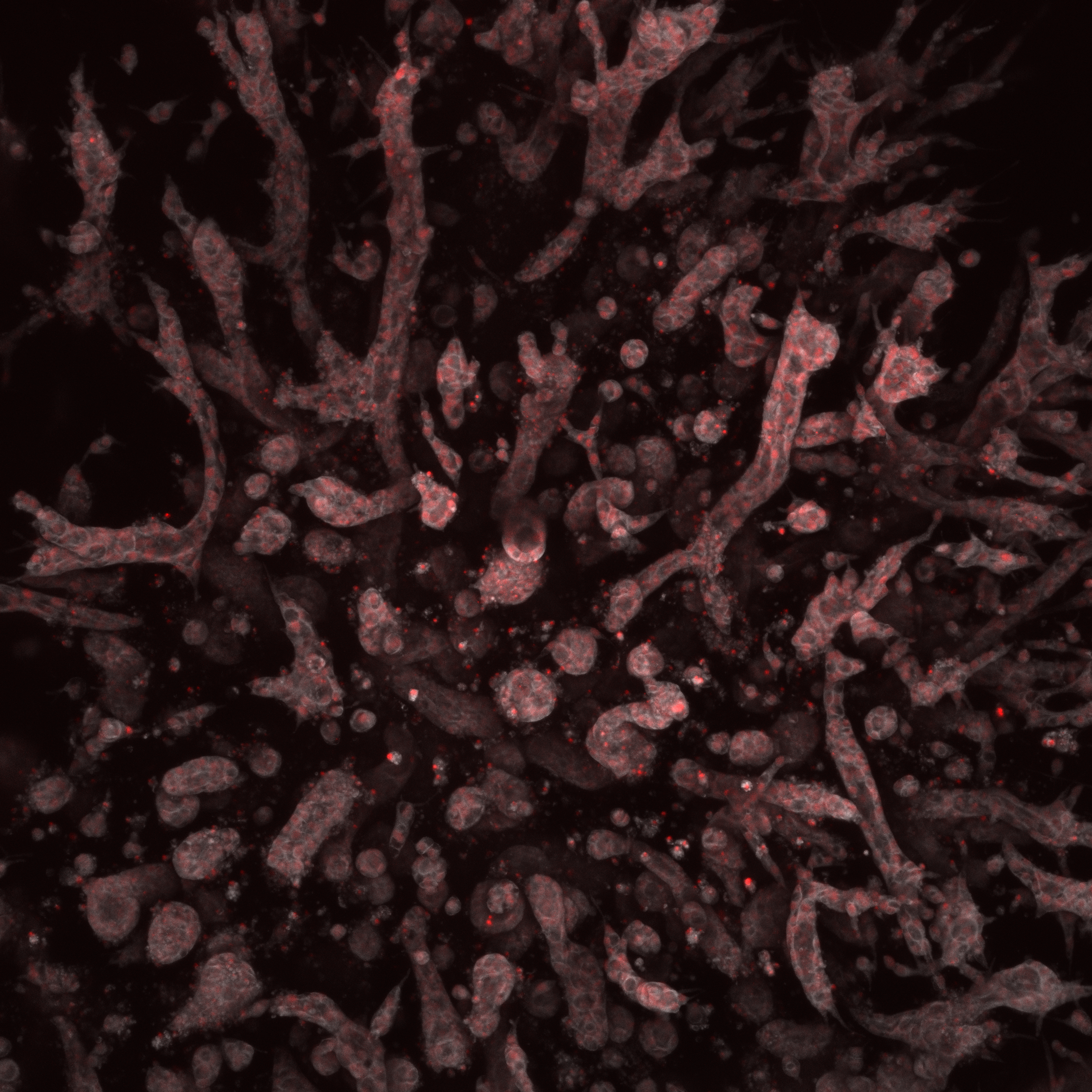

Supplement: Supplementary file 2 [file LSA-2022-01424_SdataF6.2_F7_F8_FS1_FS2_FS3_FS4_FS5.zip › HGF_Maximum intensity projection.tif]

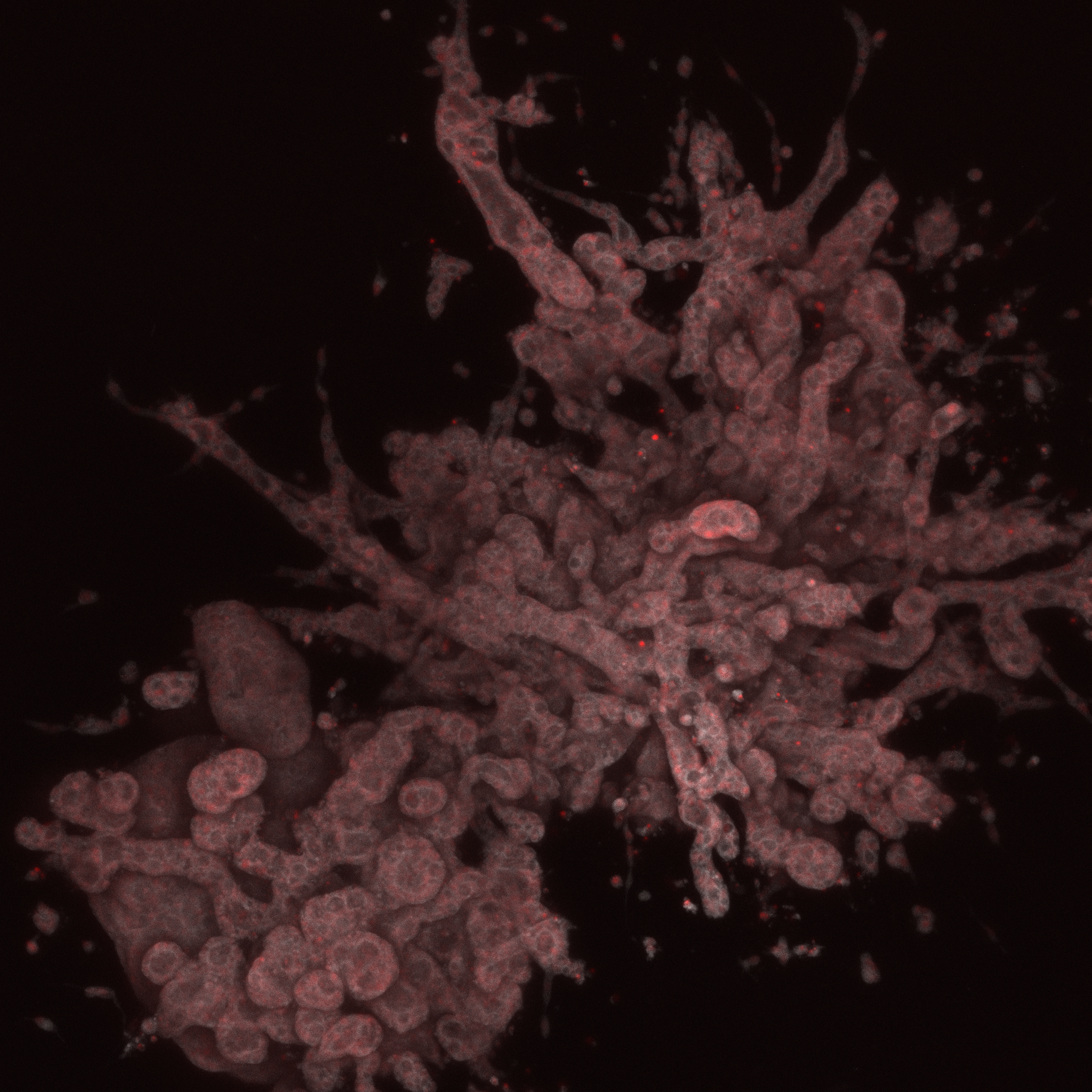

Supplement: Supplementary file 2 [file LSA-2022-01424_SdataF6.2_F7_F8_FS1_FS2_FS3_FS4_FS5.zip › HM2_Maximum intensity projection.tif]

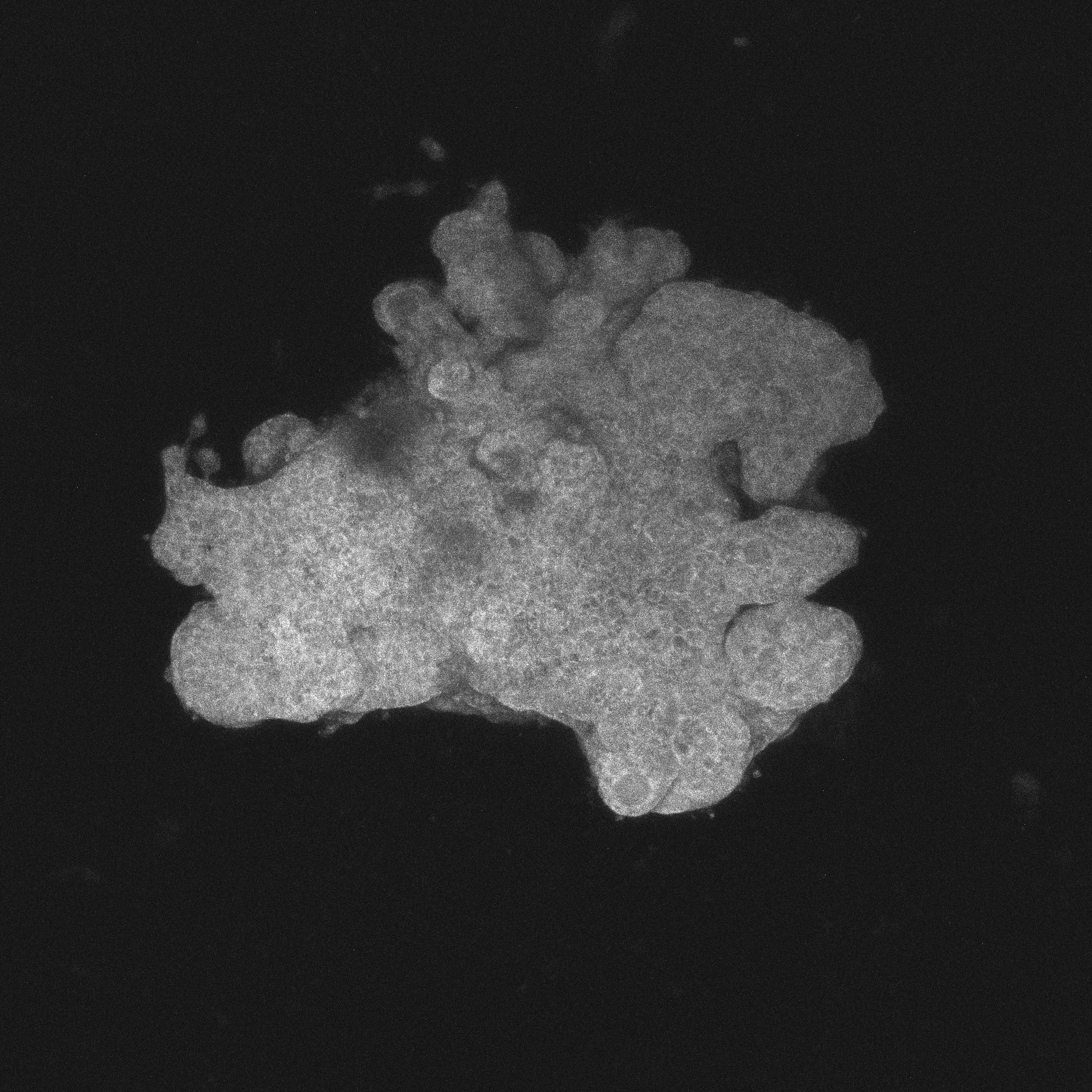

Supplement: Supplementary file 2 [file LSA-2022-01424_SdataF6.2_F7_F8_FS1_FS2_FS3_FS4_FS5.zip › HM4 x10_Maximum intensity projection.tif]

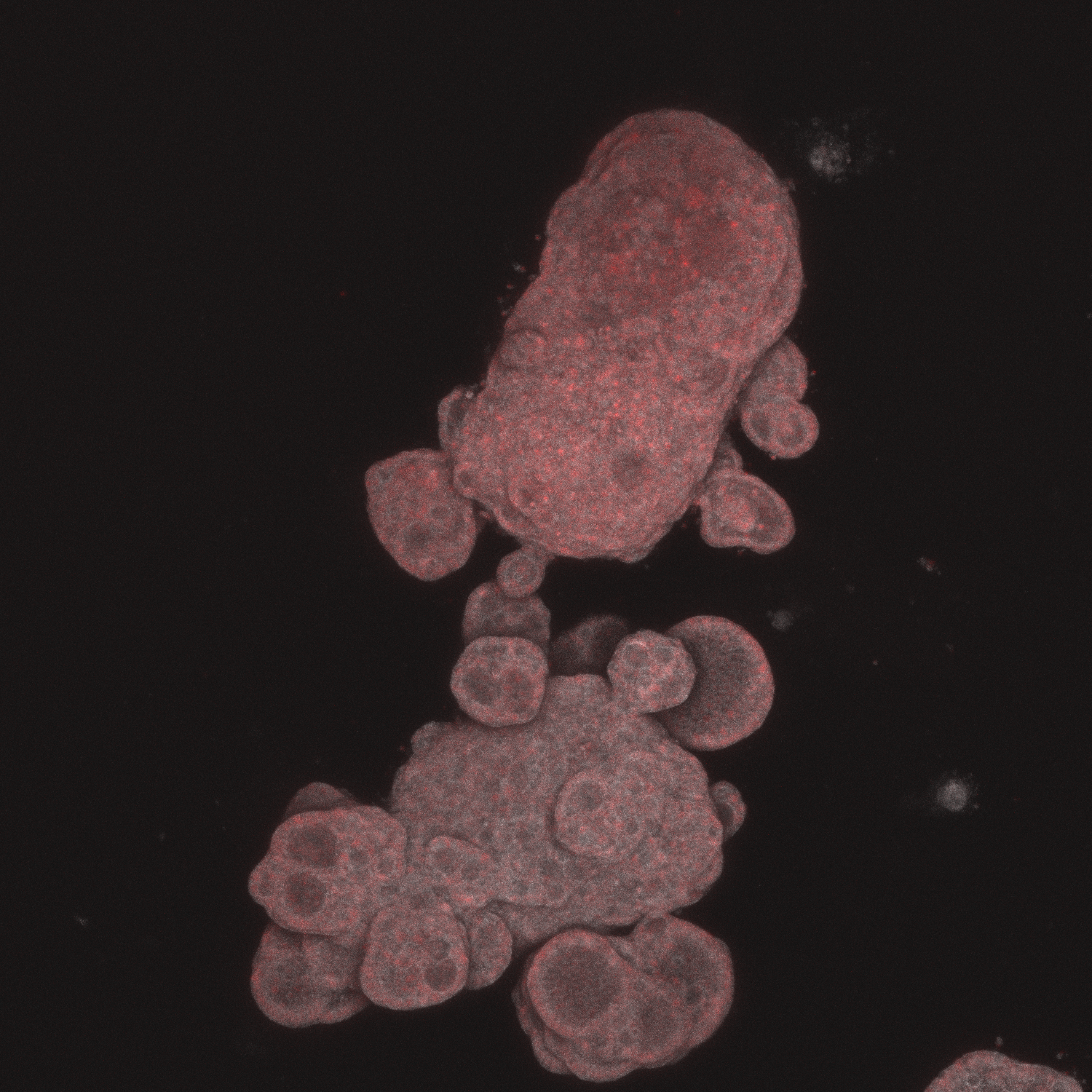

Supplement: Supplementary file 2 [file LSA-2022-01424_SdataF6.2_F7_F8_FS1_FS2_FS3_FS4_FS5.zip › HM4_Maximum intensity projection.tif]

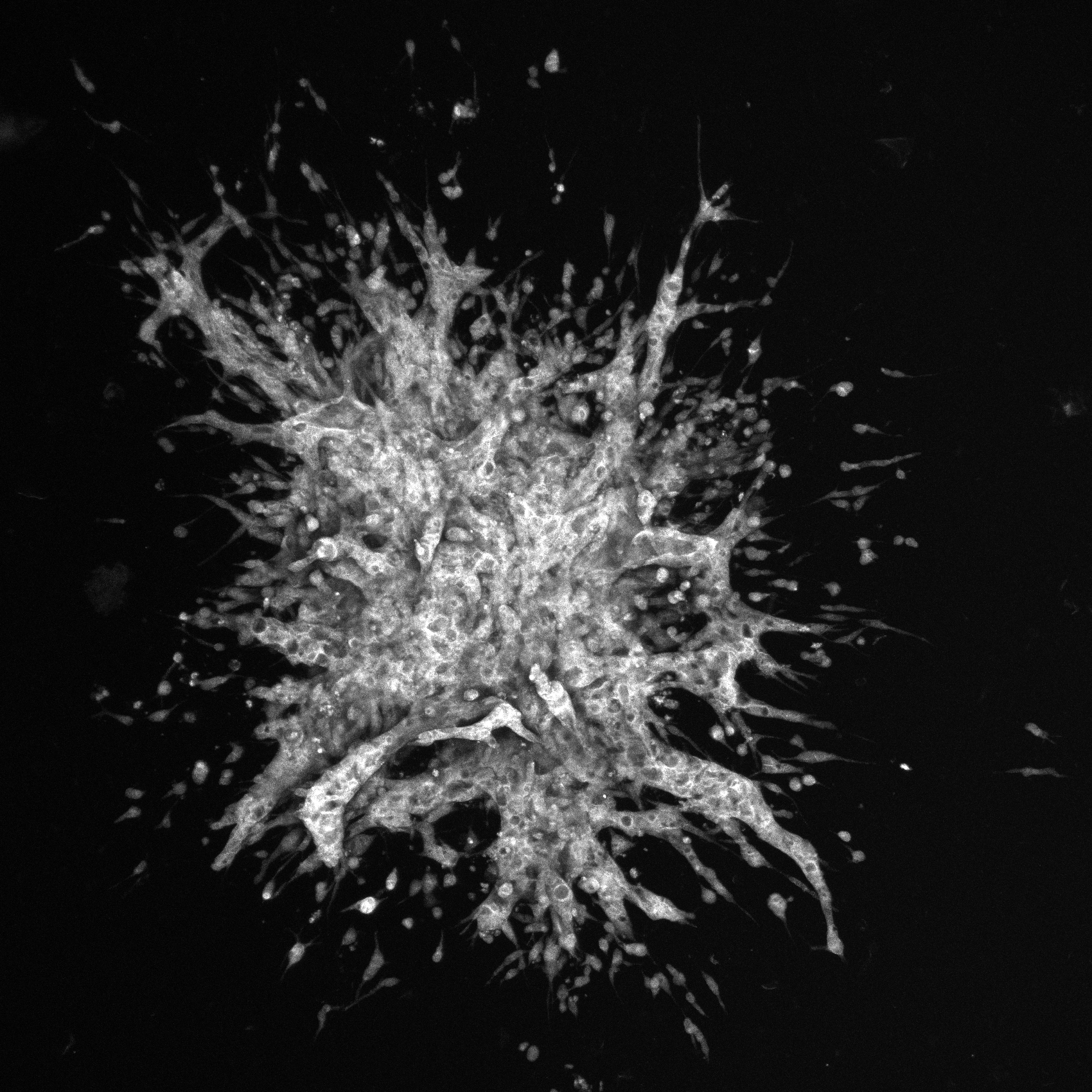

Supplement: Supplementary file 2 [file LSA-2022-01424_SdataF6.2_F7_F8_FS1_FS2_FS3_FS4_FS5.zip › K1K1 100nM_Maximum intensity projection.tif]

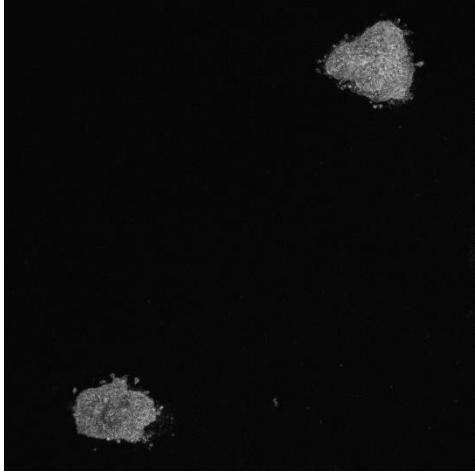

CTRL

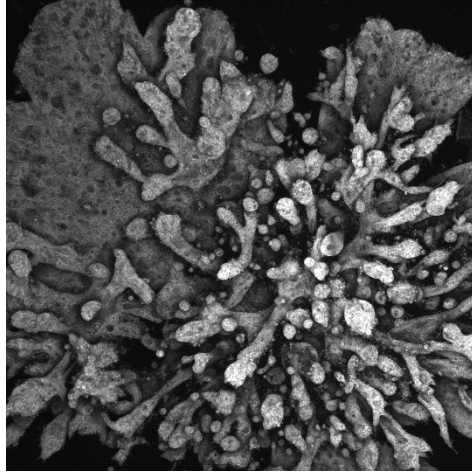

HGF 100pM

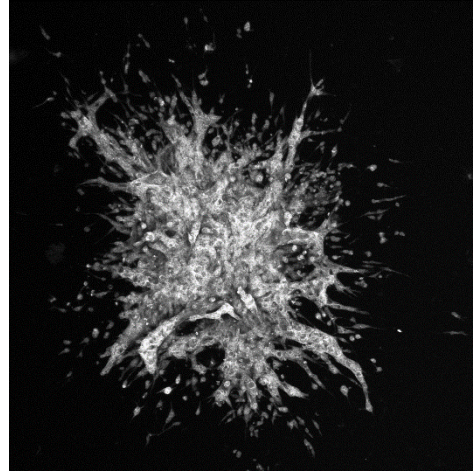

K1K1 100nM

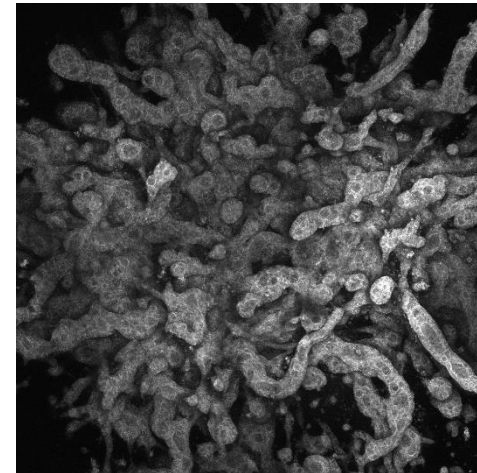

HM2 10 nM

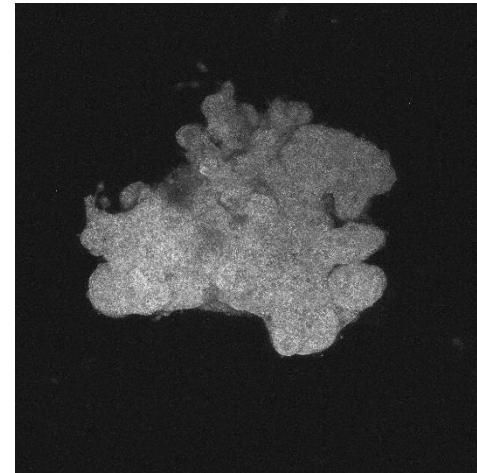

HM4 10 nM

Supplement: Supplementary file 2 [file LSA-2022-01424_SdataF6.2_F7_F8_FS1_FS2_FS3_FS4_FS5.zip › K1K1 matrigel.pdf]

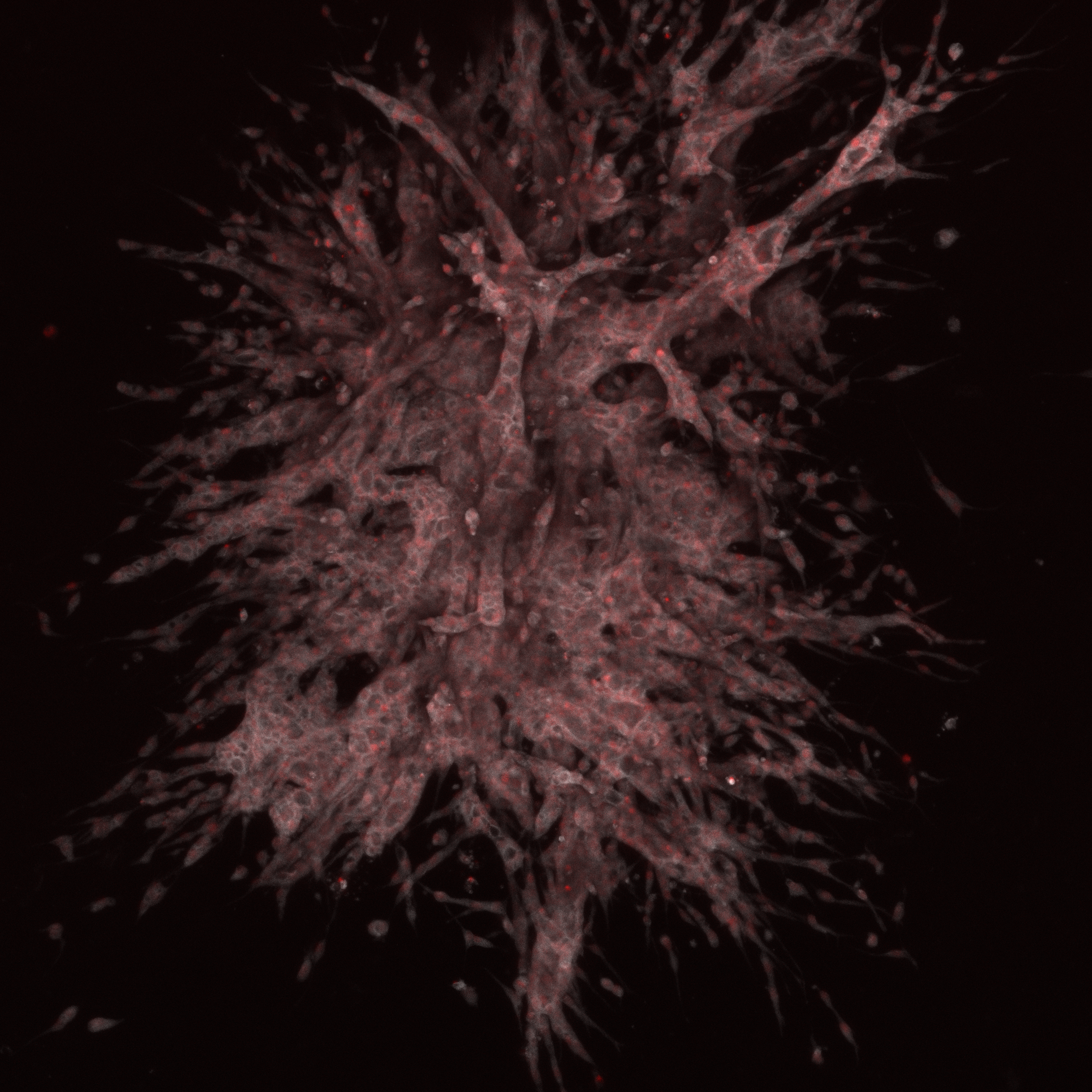

Supplement: Supplementary file 2 [file LSA-2022-01424_SdataF6.2_F7_F8_FS1_FS2_FS3_FS4_FS5.zip › K1K1_Maximum intensity projection.tif]

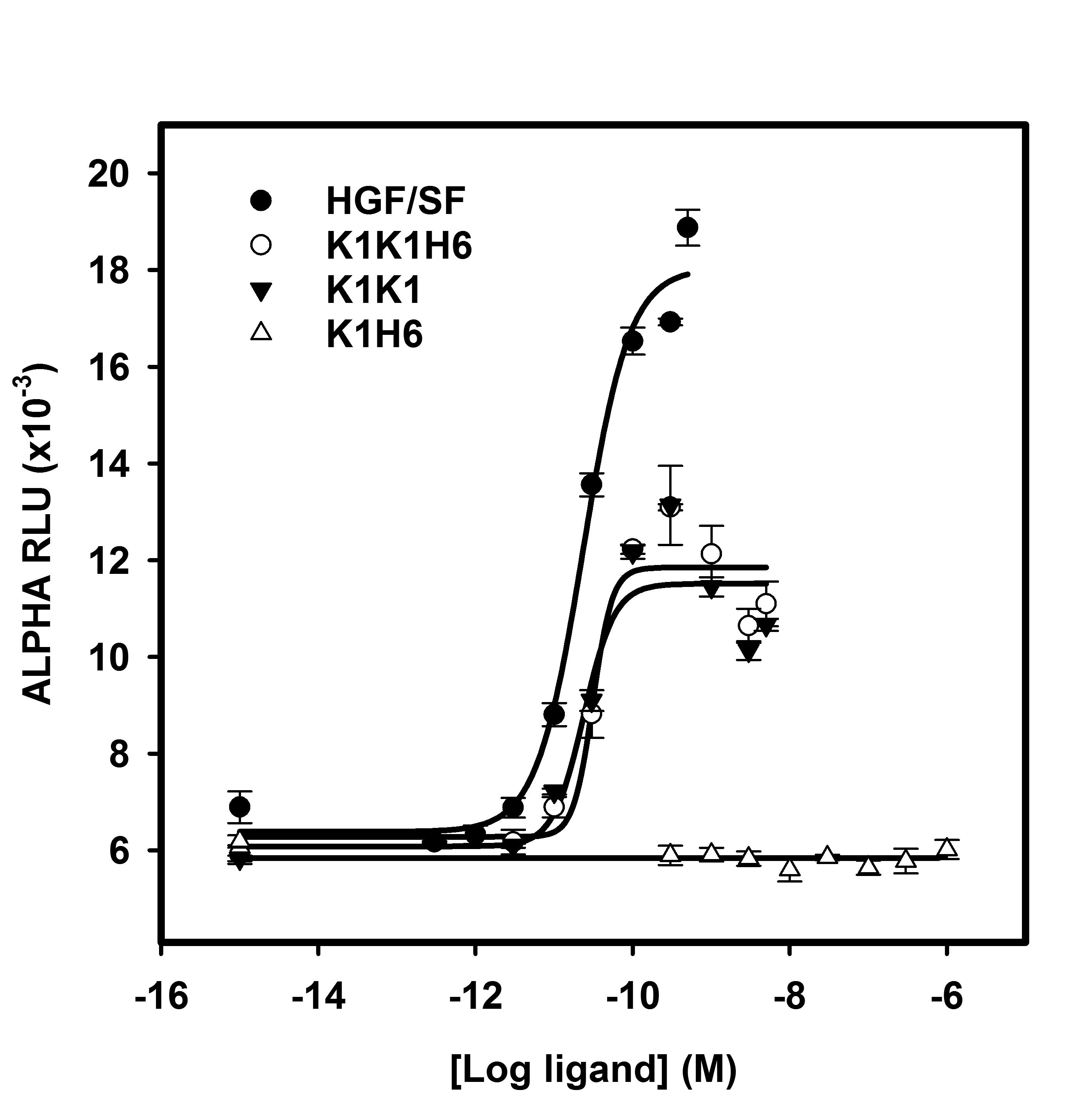

Supplement: Supplementary file 5 [file LSA-2022-01424_SdataF5.2.zip › Fig 5B Akt.JPG]

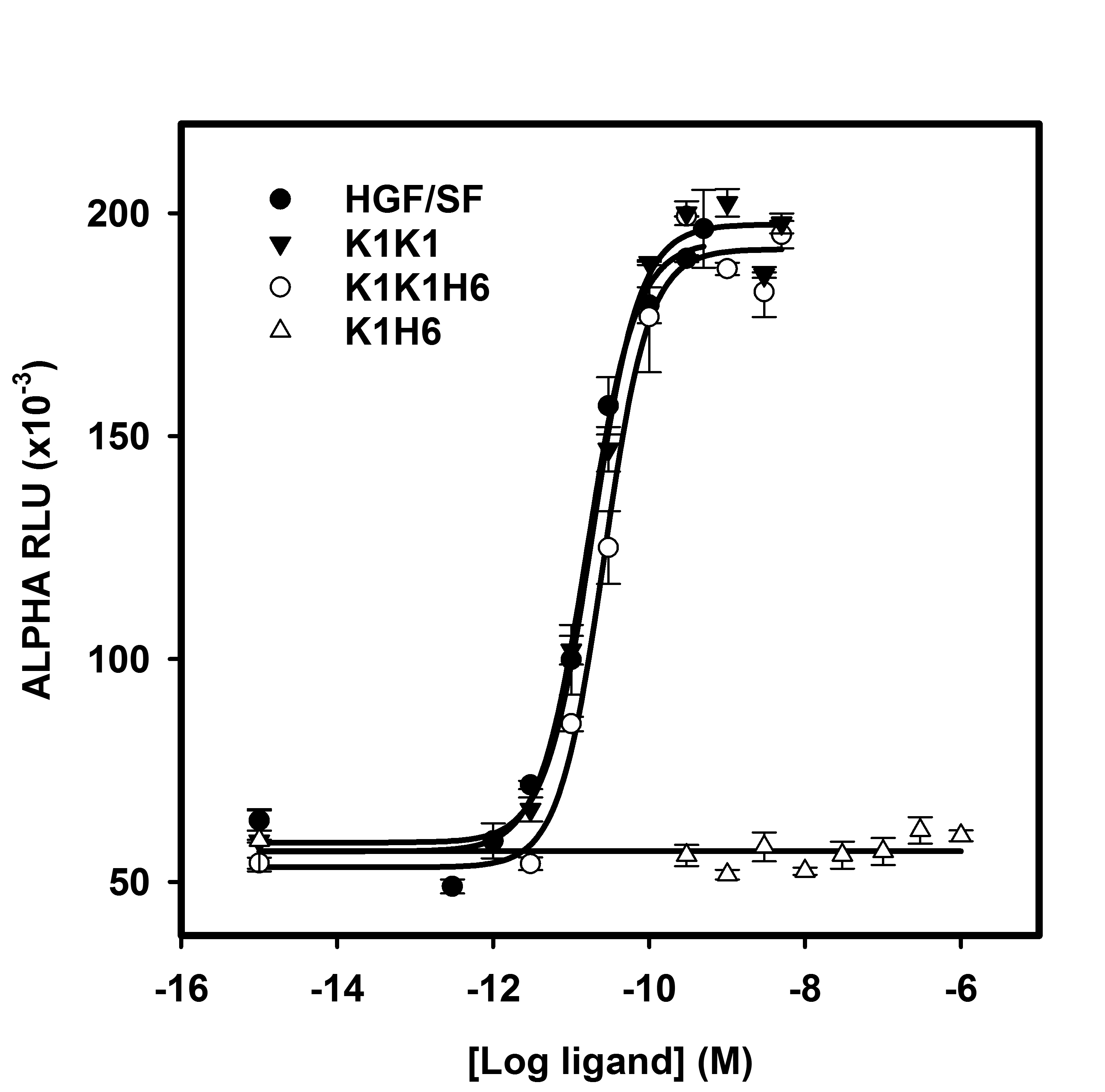

Supplement: Supplementary file 5 [file LSA-2022-01424_SdataF5.2.zip › Fig 5C ERK.JPG]

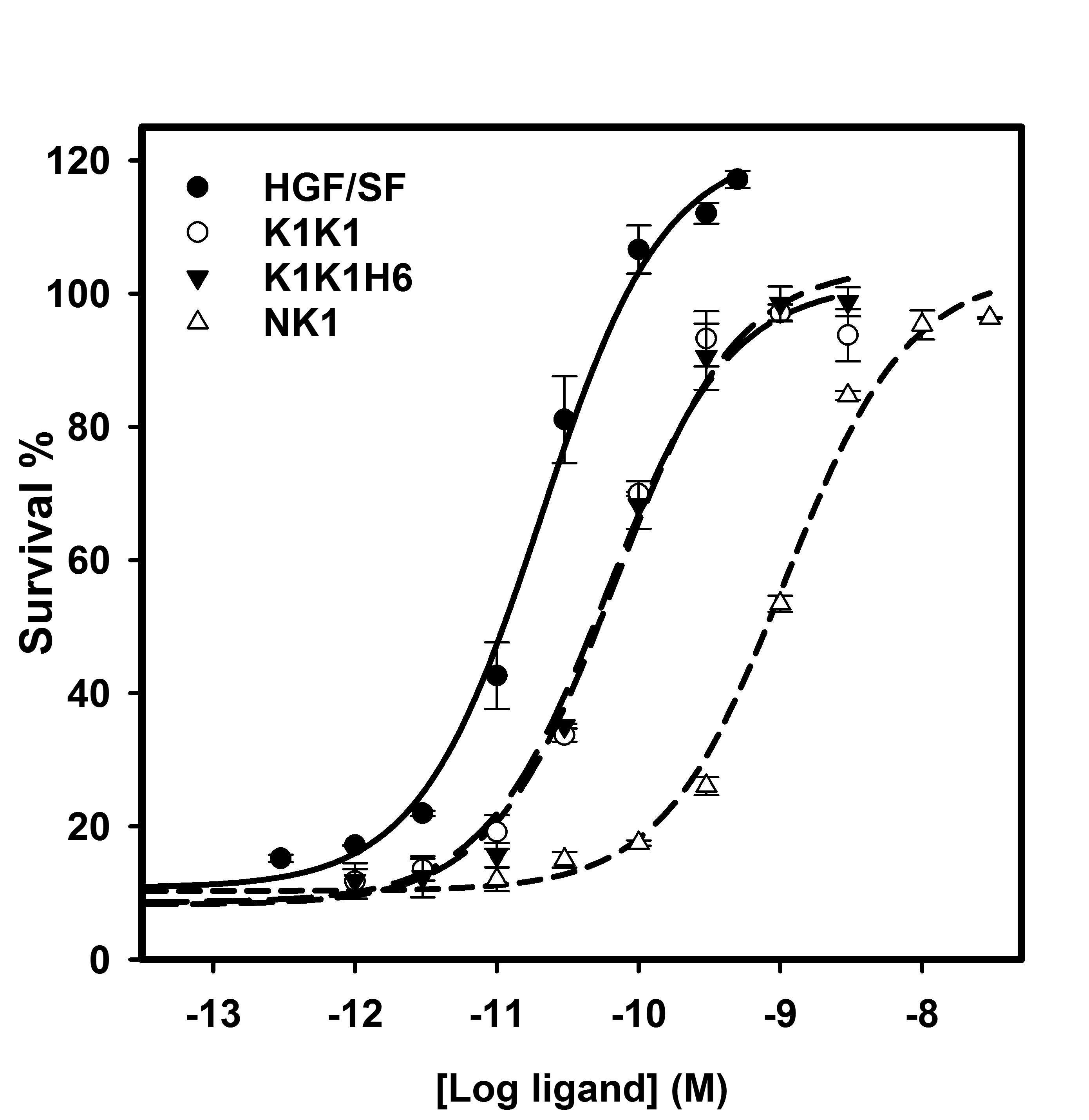

Supplement: Supplementary file 6 [file LSA-2022-01424_SdataF5.4.zip › Fig5E.JPG]

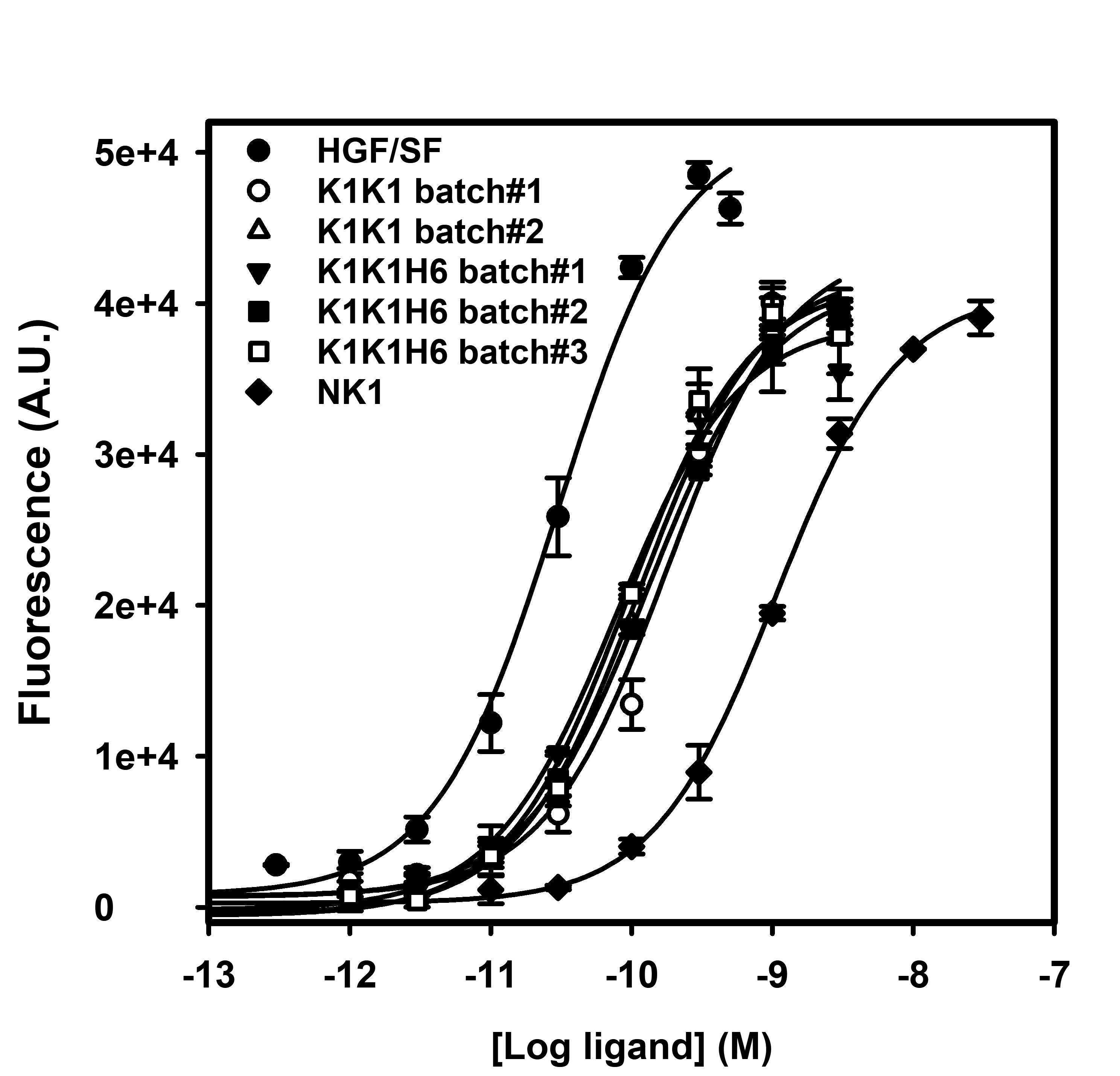

Supplement: Supplementary file 7 [file LSA-2022-01424_SdataFS4.zip › FigS4A.JPG]

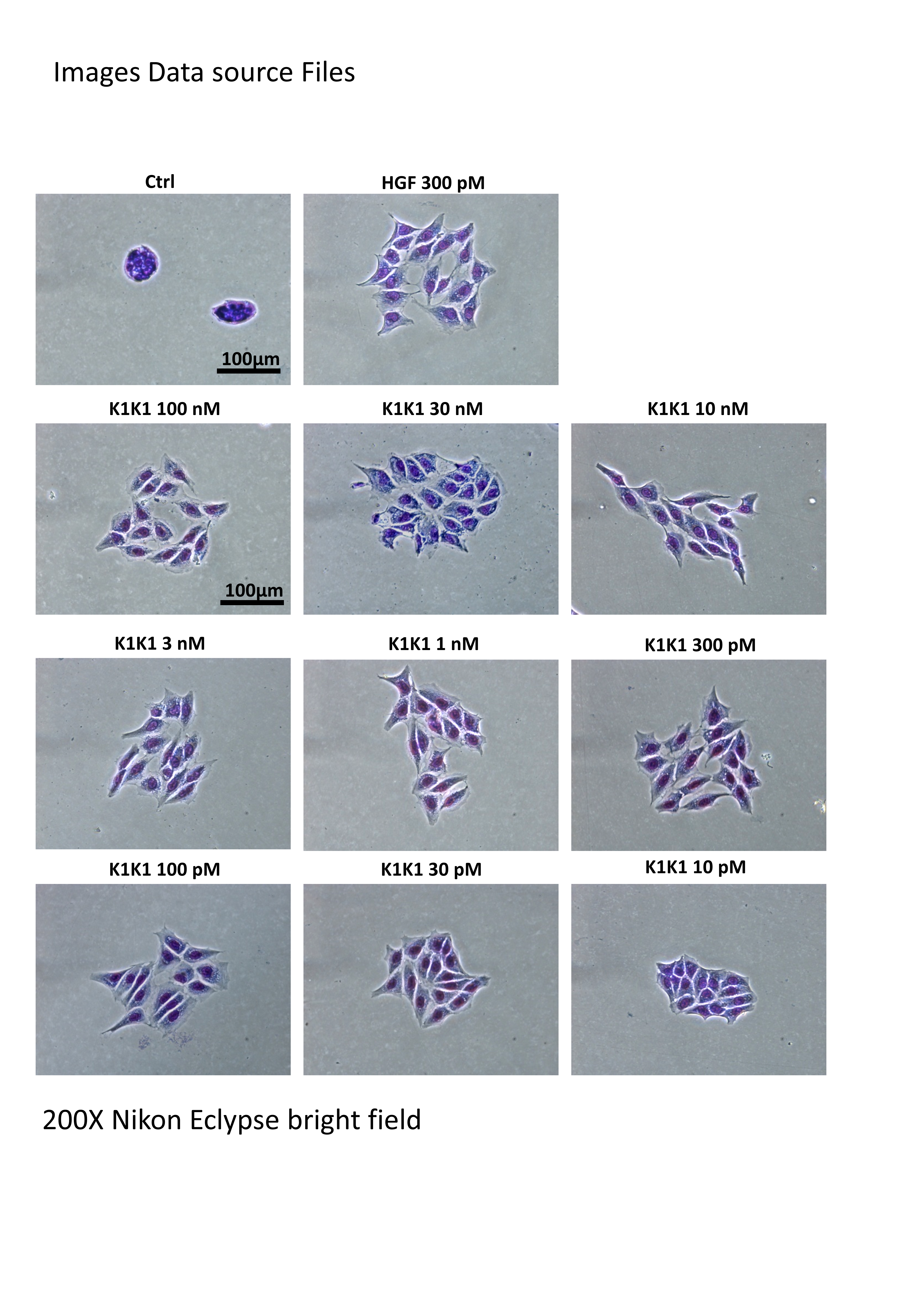

Supplement: Supplementary file 8 [file LSA-2022-01424_SdataF6.1.zip › 200X Nikon Eclypse bright field_MDCK_A.tif]

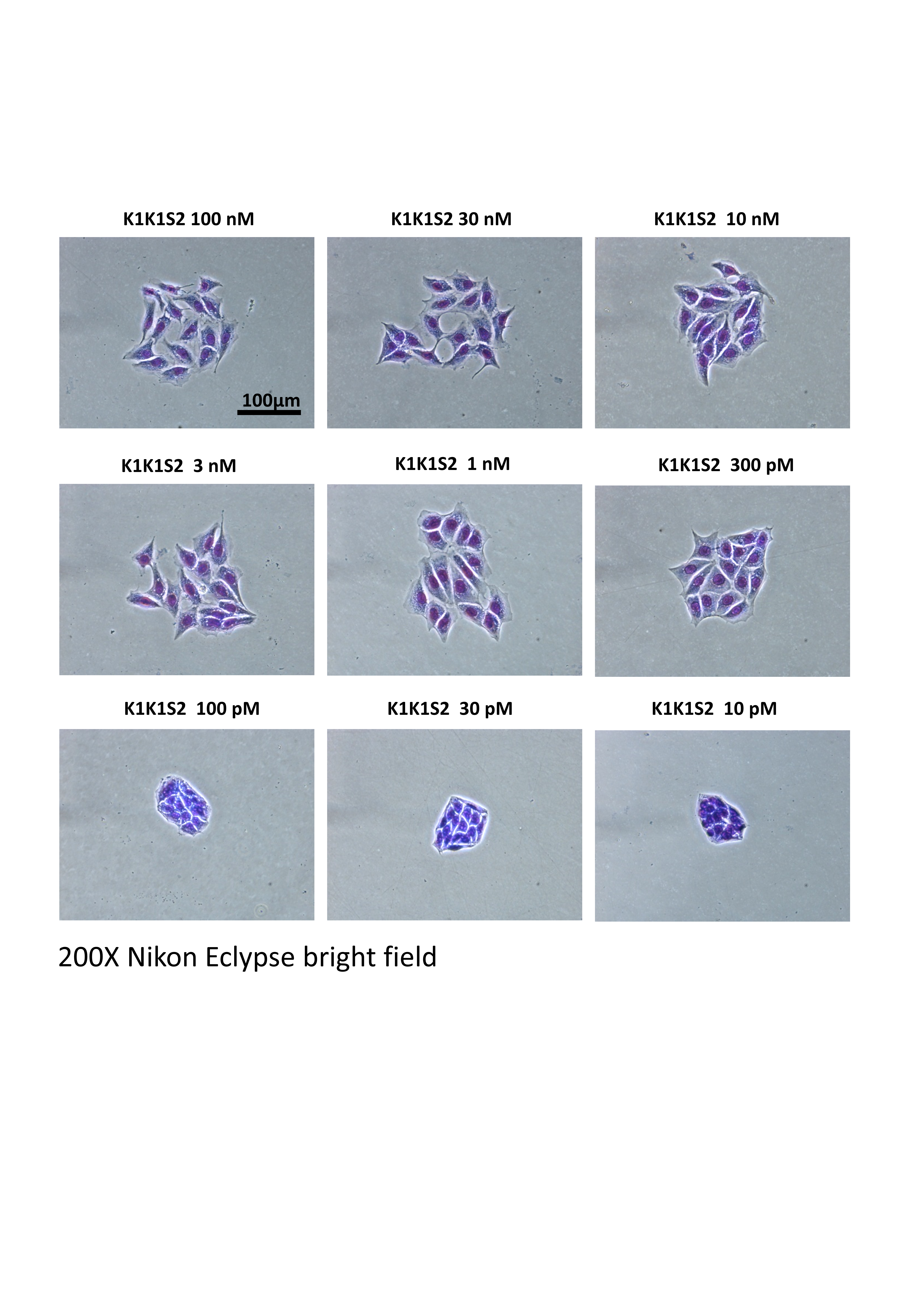

Supplement: Supplementary file 8 [file LSA-2022-01424_SdataF6.1.zip › 200X Nikon Eclypse bright field_MDCK_B.tif]

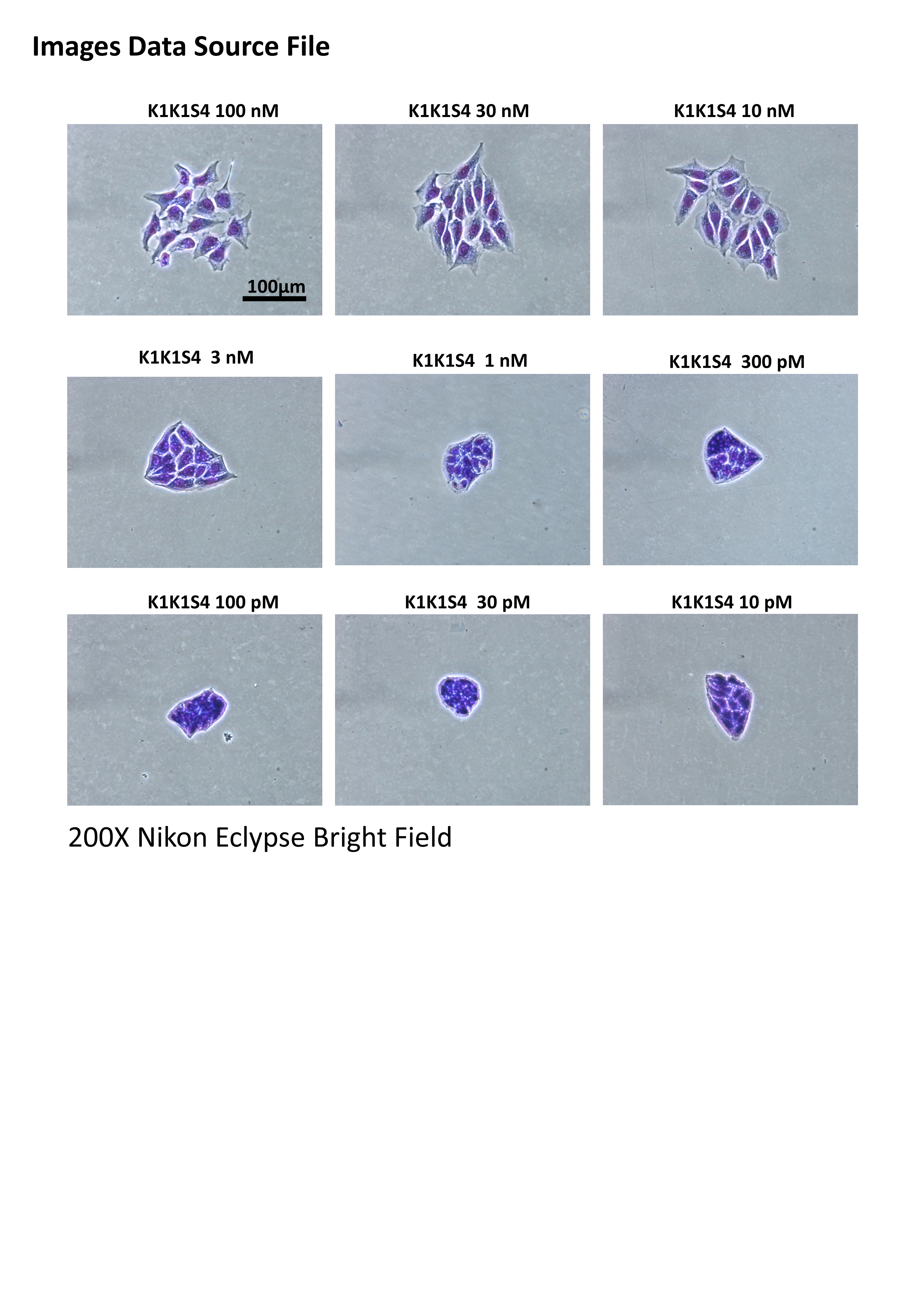

Supplement: Supplementary file 8 [file LSA-2022-01424_SdataF6.1.zip › 200X Nikon Eclypse bright field_MDCK_C.tif]

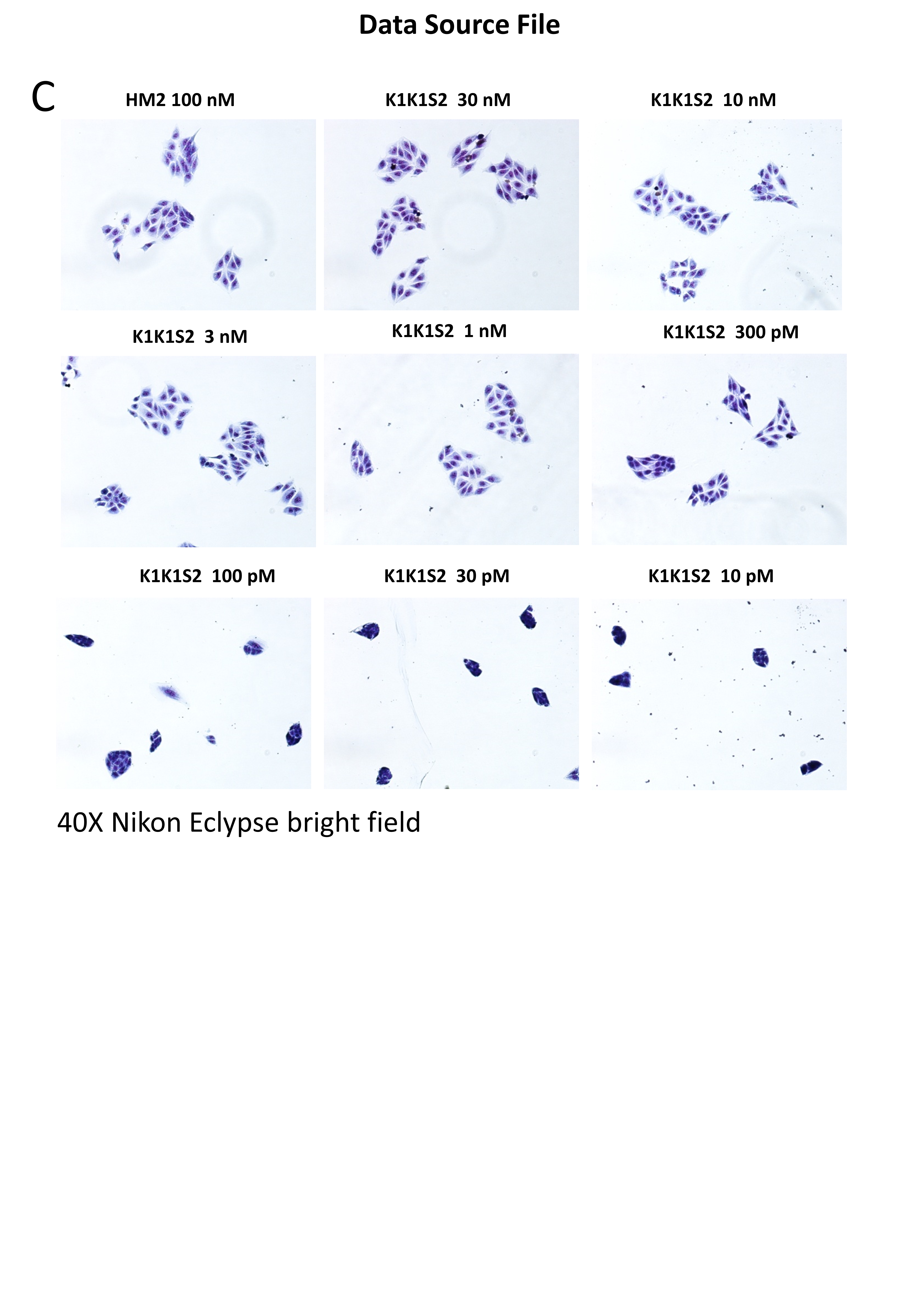

Supplement: Supplementary file 8 [file LSA-2022-01424_SdataF6.1.zip › 40X Nikon Eclypse bright field_MCDK_B.tif]

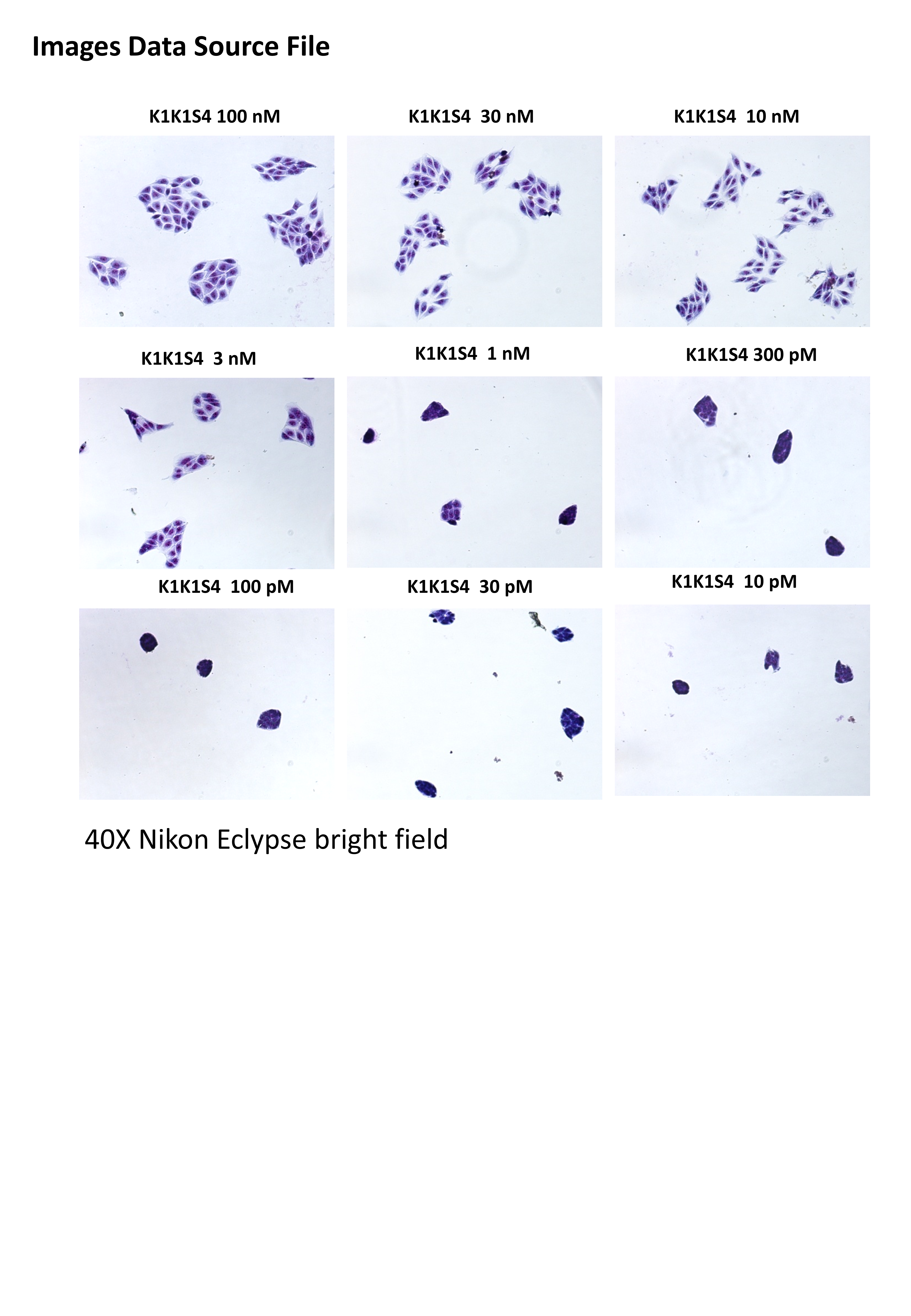

Supplement: Supplementary file 8 [file LSA-2022-01424_SdataF6.1.zip › 40X Nikon Eclypse bright field_MCDK_C.tif]

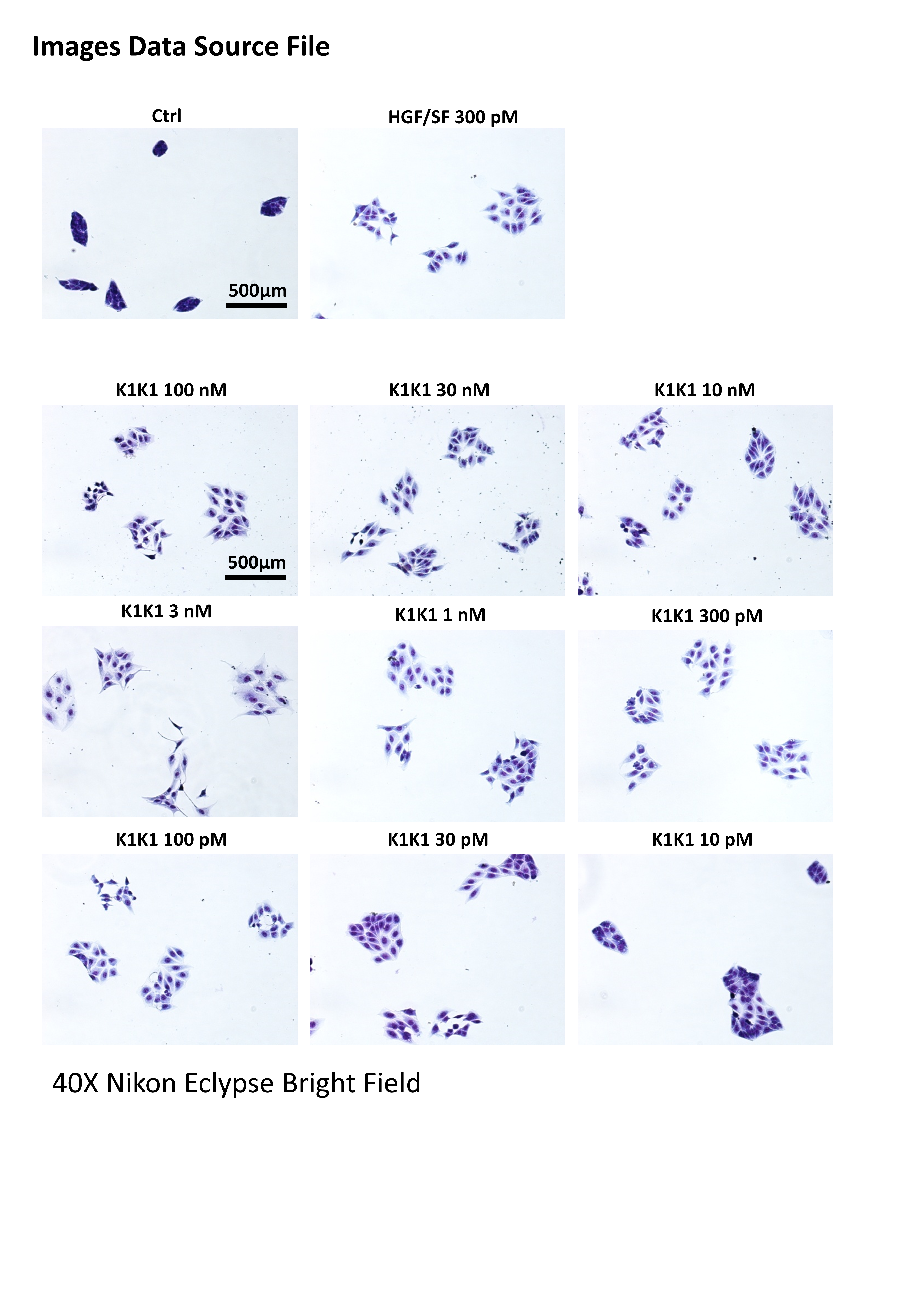

Supplement: Supplementary file 8 [file LSA-2022-01424_SdataF6.1.zip › 40X Nikon Eclypse bright field_MDCK_A.tif]

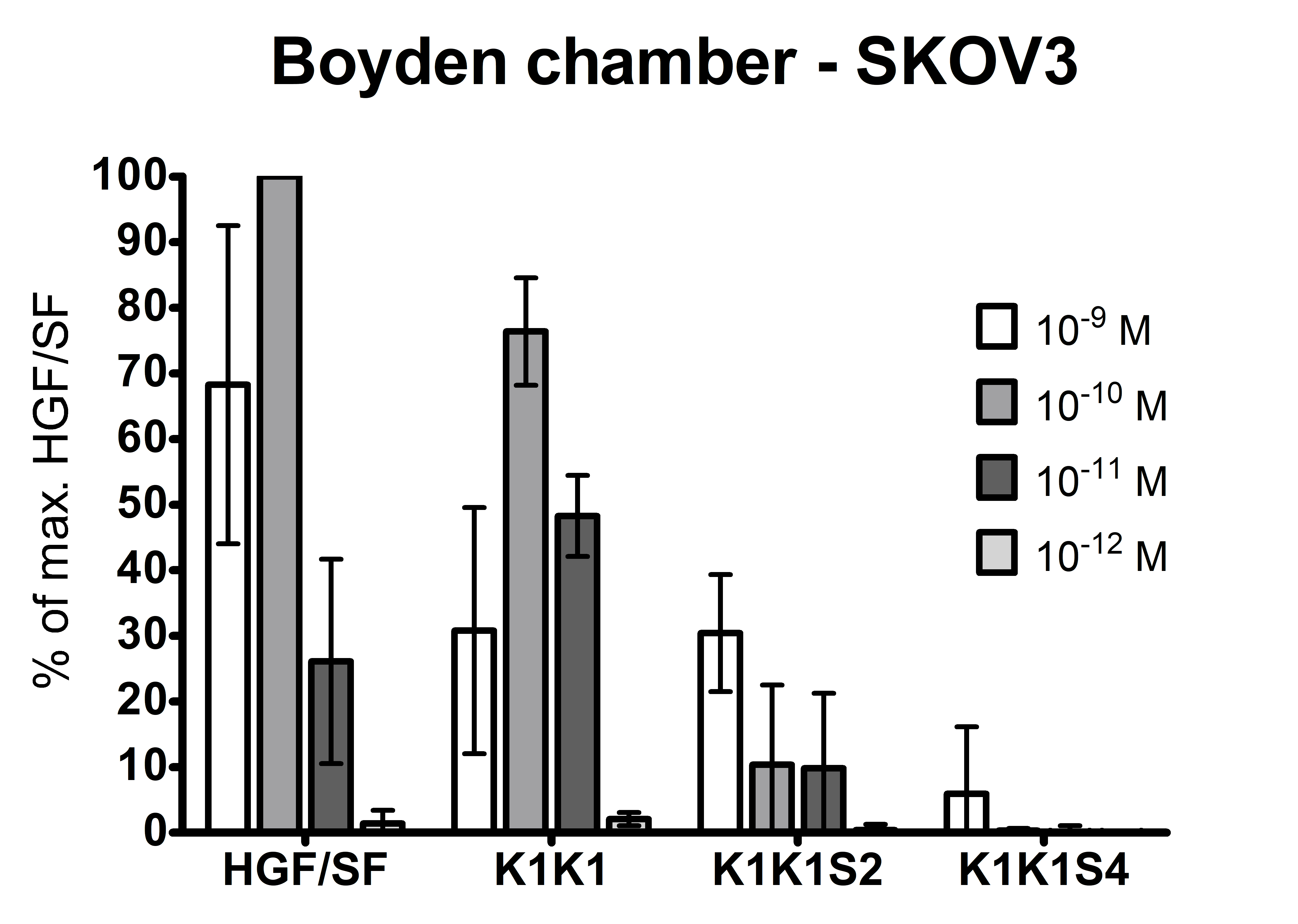

Supplement: Supplementary file 9 [file LSA-2022-01424_SdataF6.3.zip › per ligand.tif]

Fig. S5C

Digitalized images (LAS3000 system) of blotted membranes

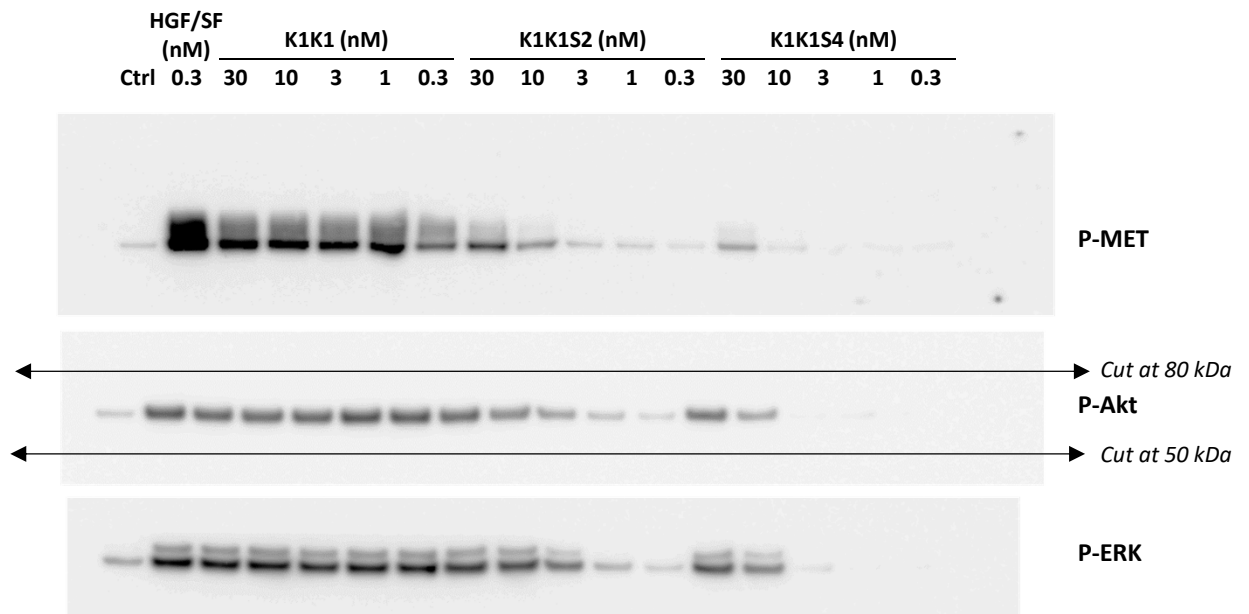

Membranes stripped and reprobed

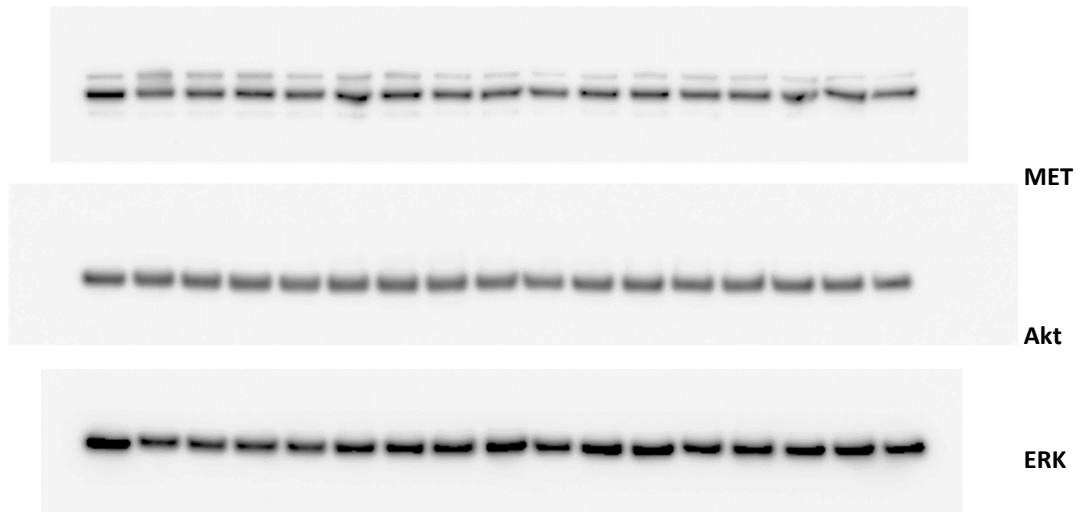

Supplement: Supplementary file 10 [file LSA-2022-01424_SdataFS5.pdf]

Fig. S6

Digitalized images (LAS3000 system) of blotted membranes

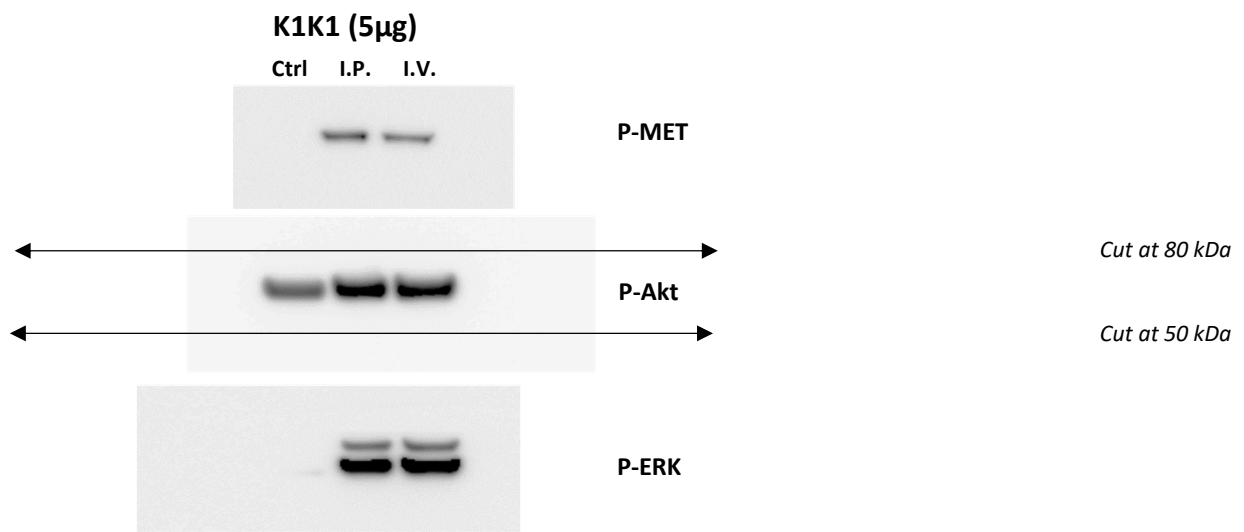

Membranes stripped and reprobed

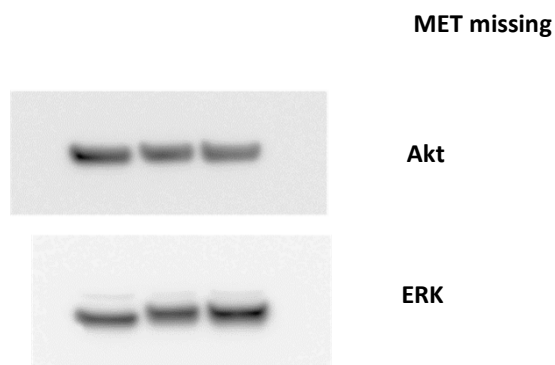

Supplement: Supplementary file 11 [file LSA-2022-01424_SdataFS6.pdf]
