# Supplementary material for: Dimerization of kringle 1 domain from hepatocyte growth factor/scatter factor provides a potent MET receptor agonist
Source: Life Sci Alliance. 2022 Jul 29;5(12):e202201424. doi: 10.26508/lsa.202201424 (PMC9348577; doi:10.26508/lsa.202201424)
Supplement: Supplementary file 2 [file LSA-2022-01424_SdataF6.2_F7_F8_FS1_FS2_FS3_FS4_FS5.zip › K1K1 matrigel + DAPI.pdf]

Staining:
Evans Blue 1/10000 (grey)
Ex 488 Em 508-695
DAPI 2nM (red)
Ex 395 Em 410-490
LSM 880 Zeiss
Z-stack (14.8µ)
Max Intensity Projection

CTRL

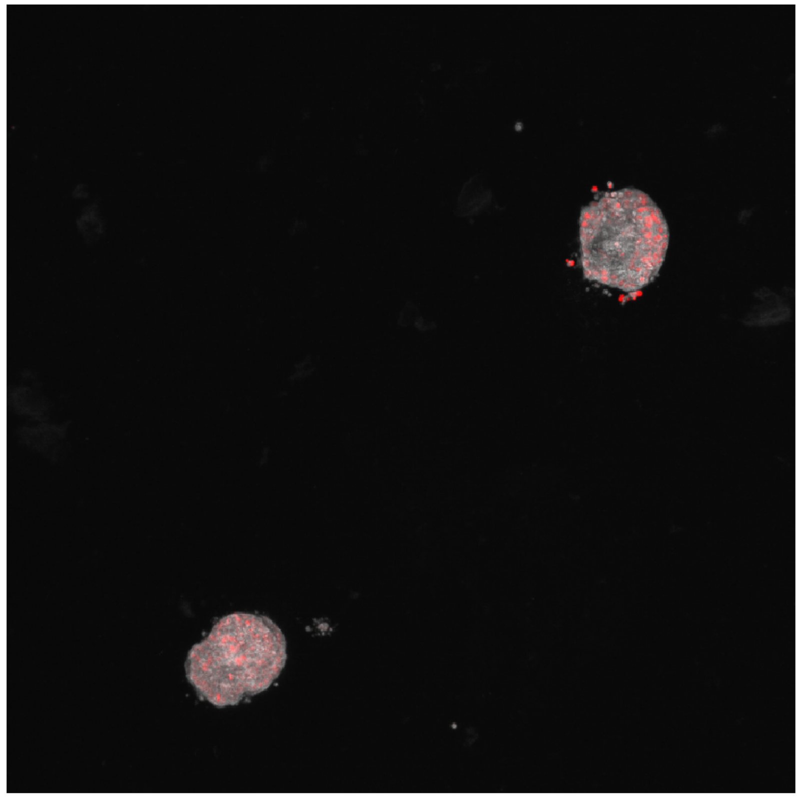

HGF 100pM

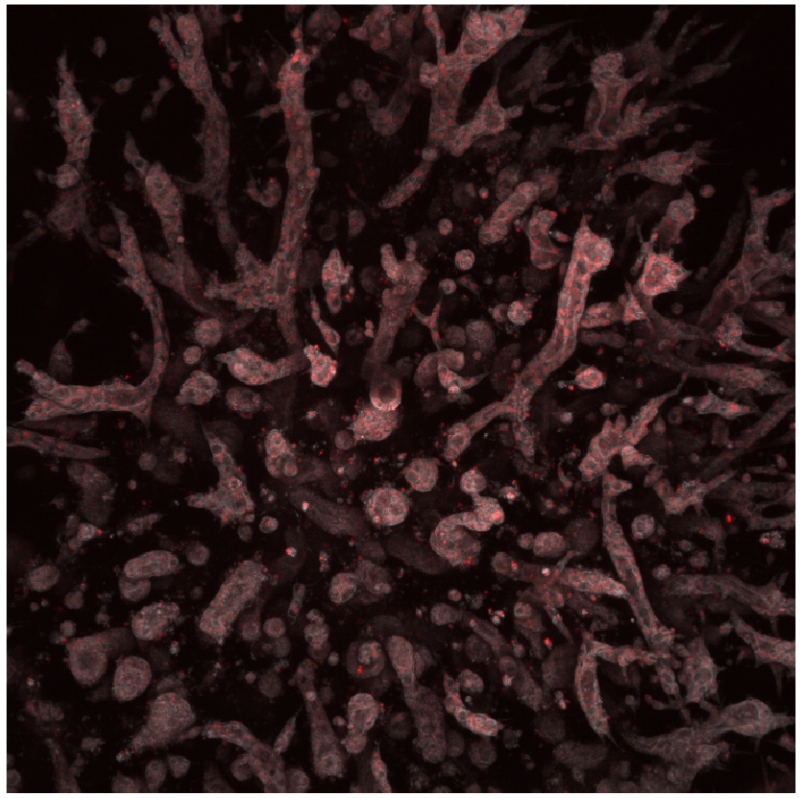

MDCK Cells
Seeded in Collagen/matrigel
Mixture
4 weeks growth in
DMEM/10% FBS
Twice a week treatments

4% PAF/PBS fixation

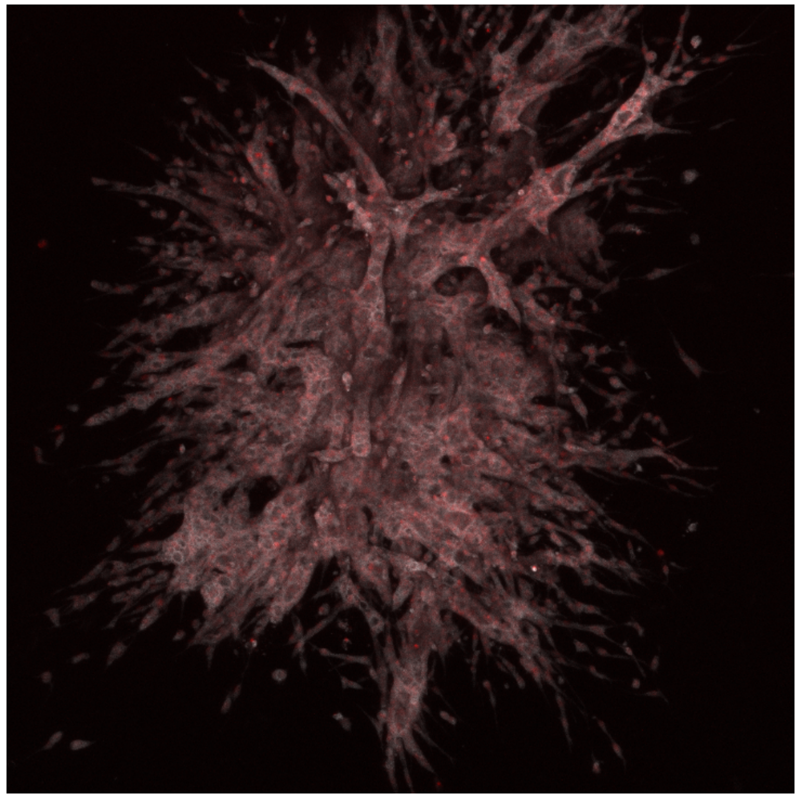

K1K1 10nM

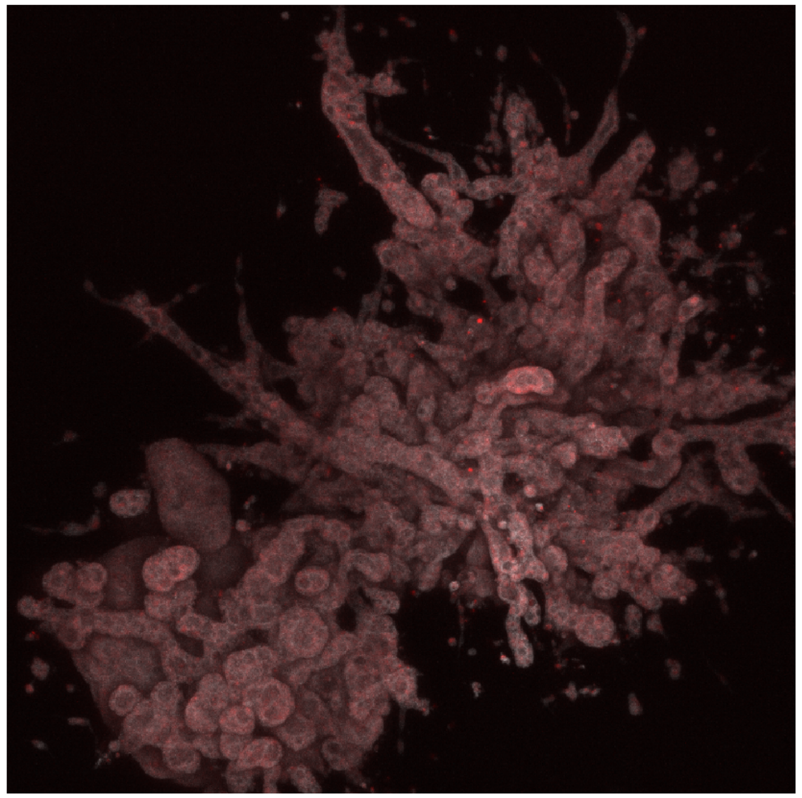

HM2 10nM

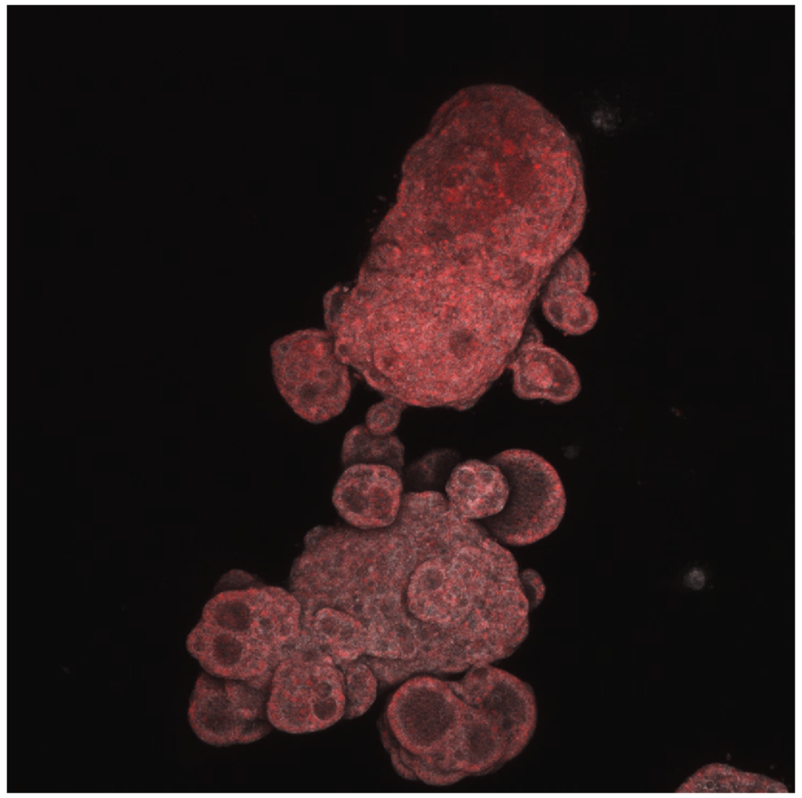

HM4 10nM
