# Supplementary material for: Dimerization of kringle 1 domain from hepatocyte growth factor/scatter factor provides a potent MET receptor agonist
Source: Life Sci Alliance. 2022 Jul 29;5(12):e202201424. doi: 10.26508/lsa.202201424 (PMC9348577; doi:10.26508/lsa.202201424)

Fig. 5A

Full scan of argentic films of blotted membranes after reassembly

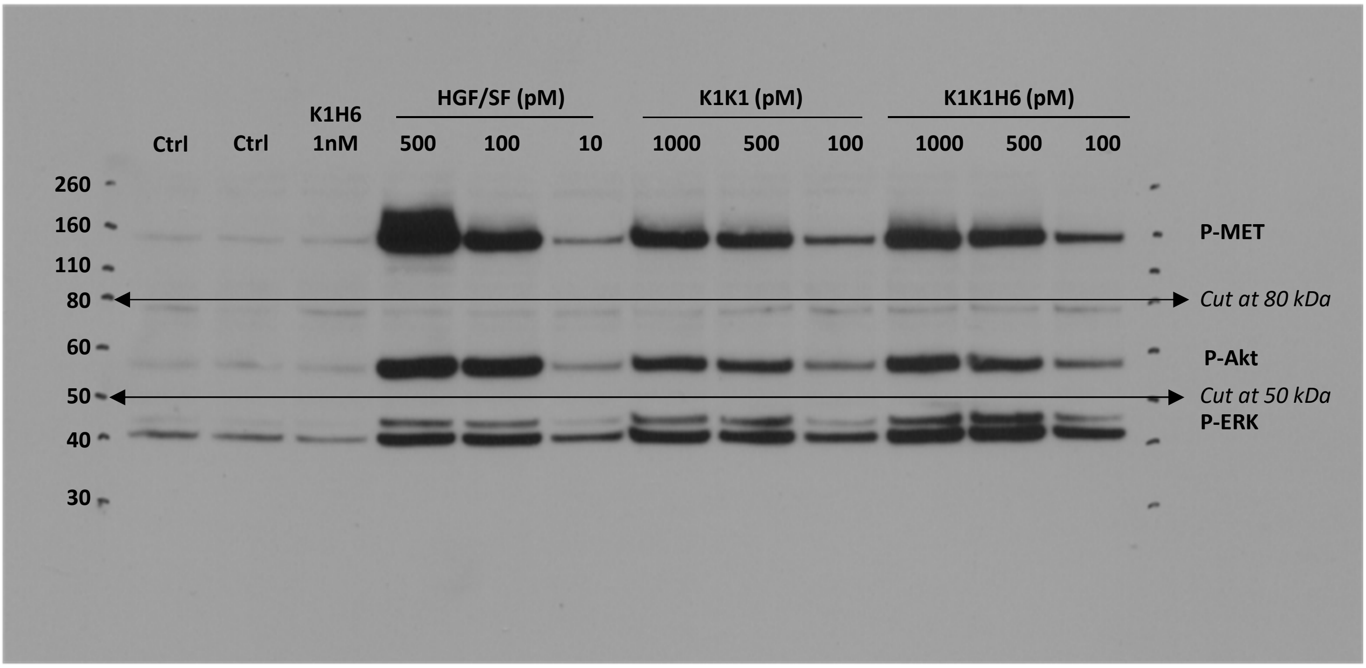

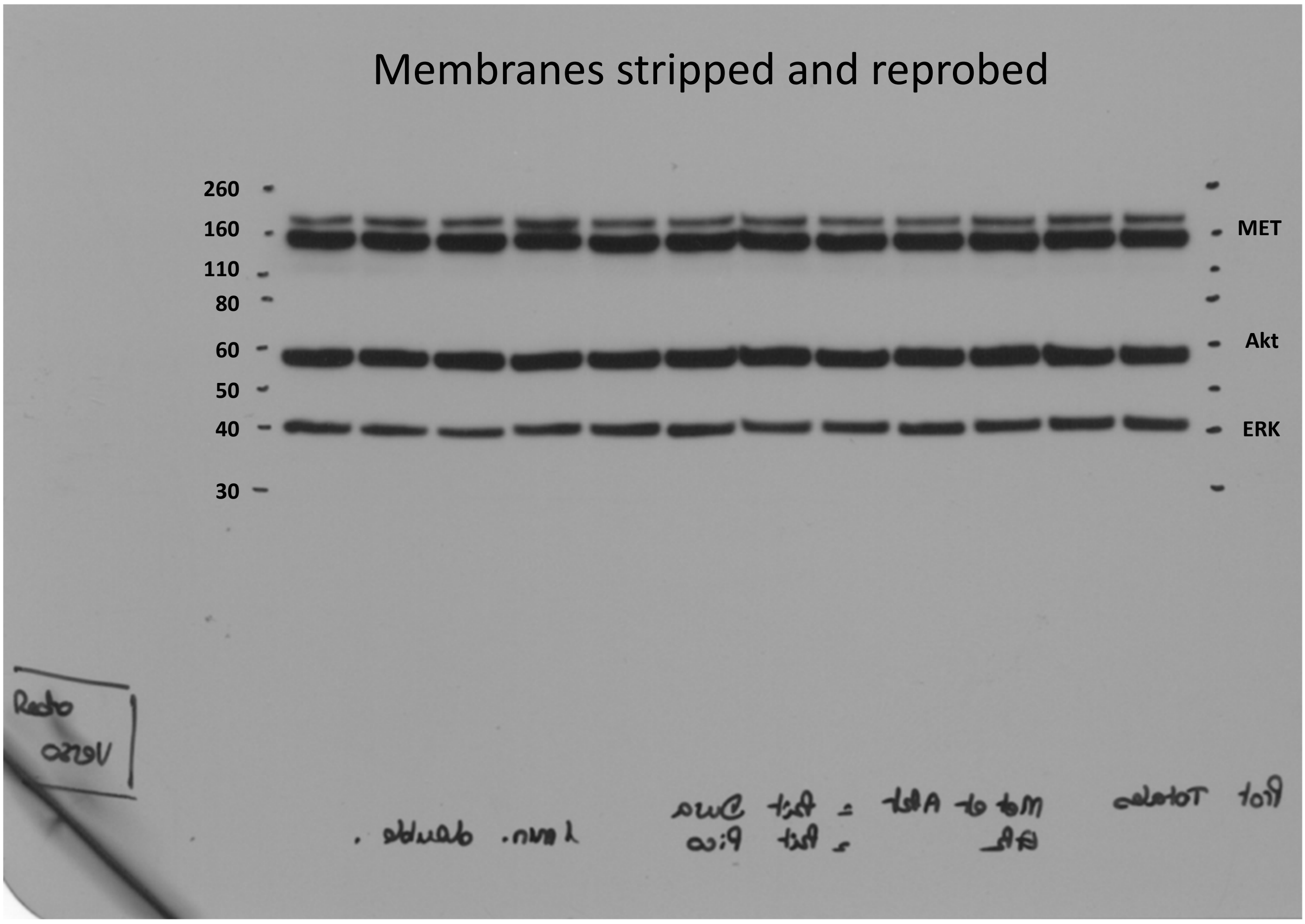

Supplement: Supplementary file 4 [file LSA-2022-01424_SdataF5.1.pdf]
