# Supplementary material for: Dimerization of kringle 1 domain from hepatocyte growth factor/scatter factor provides a potent MET receptor agonist
Source: Life Sci Alliance. 2022 Jul 29;5(12):e202201424. doi: 10.26508/lsa.202201424 (PMC9348577; doi:10.26508/lsa.202201424)
Supplement: Supplementary file 8 [file LSA-2022-01424_SdataF6.1.zip › SourceDataForFigure6A.pdf]

Images Data Source File

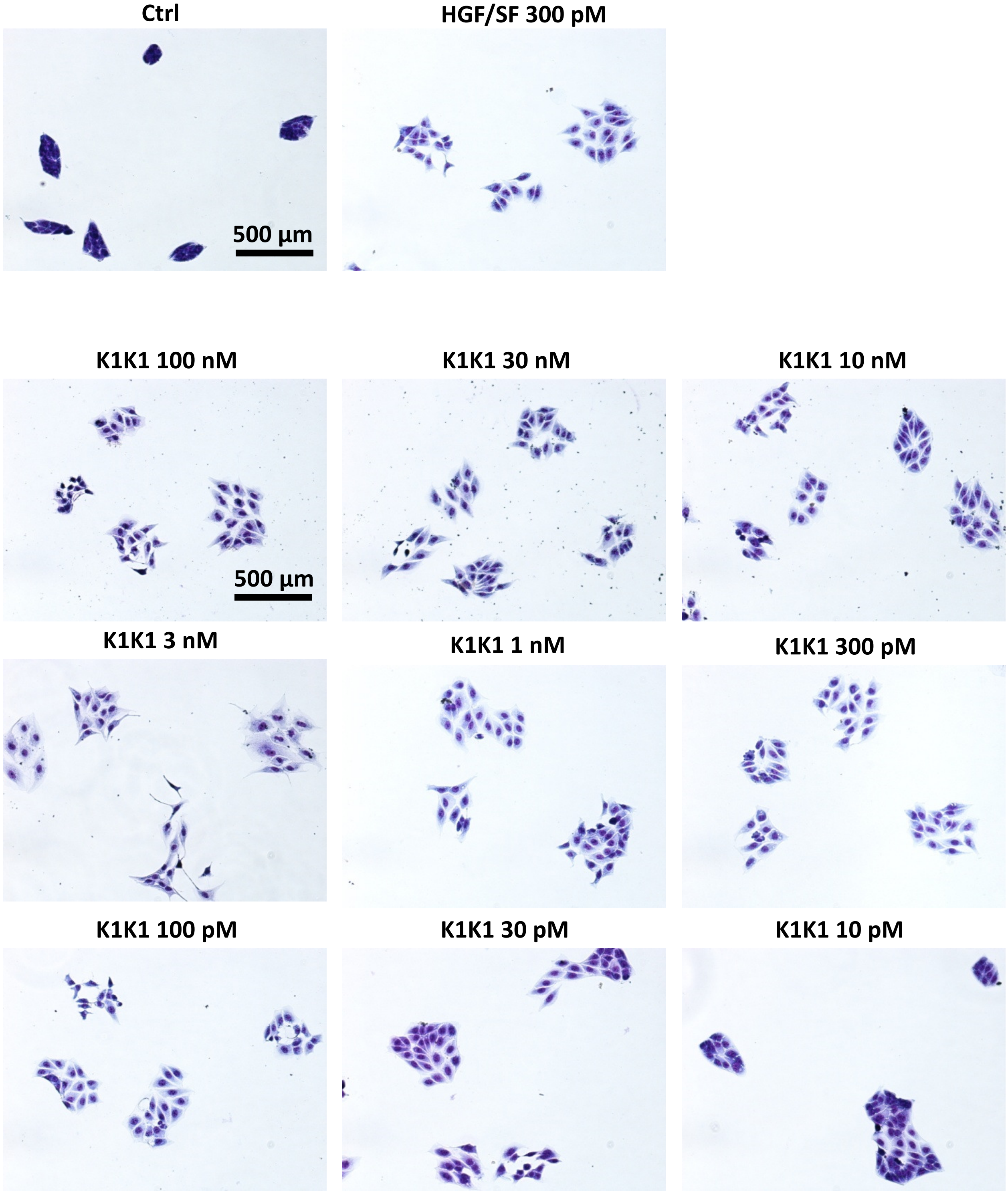

40X Nikon Eclypse bright field

Images Data Source File

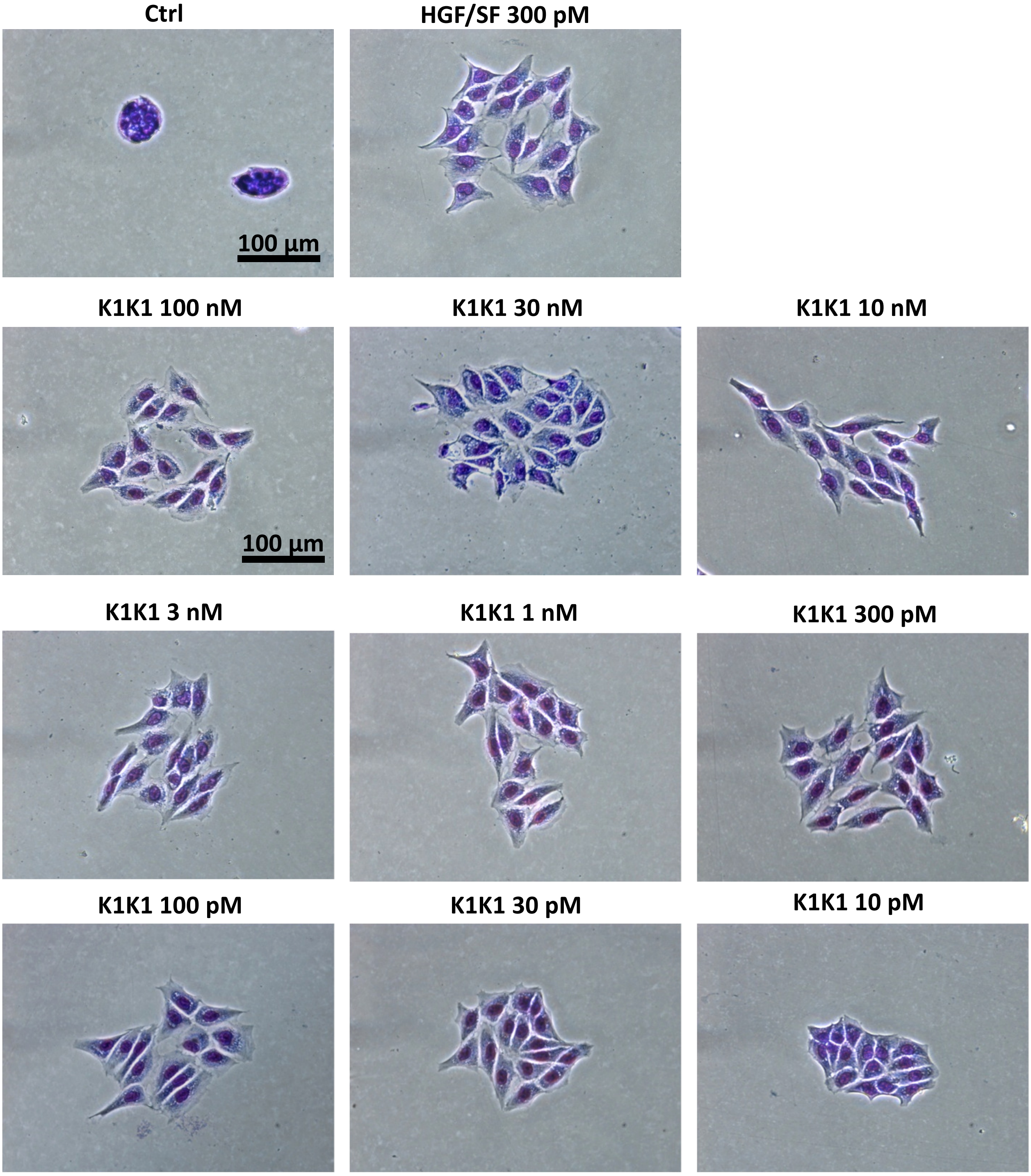

200X Nikon Eclypse bright field

Images Data Source File

HM2 100 nM

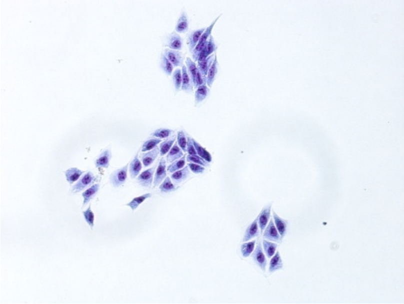

K1K1S2 30 nM

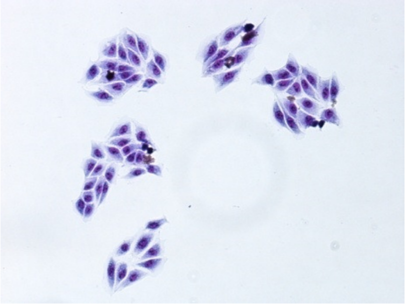

K1K1S2 10 nM

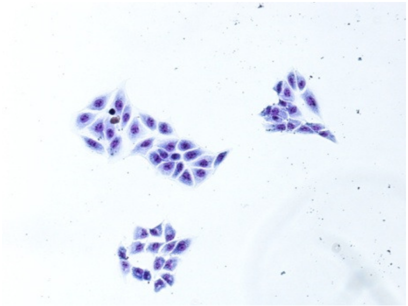

K1K1S2 3 nM

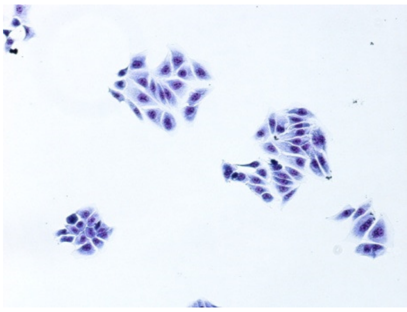

K1K1S2 1 nM

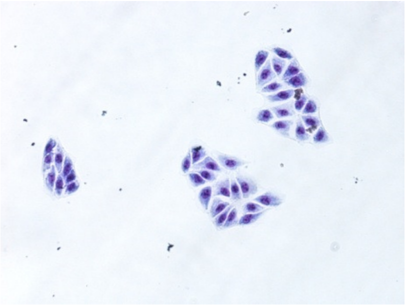

K1K1S2 300 pM

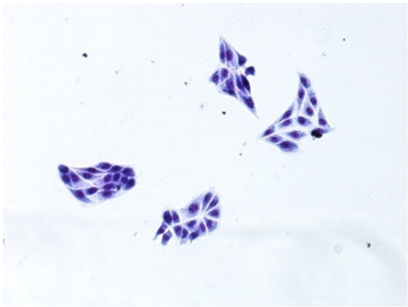

K1K1S2 100 pM

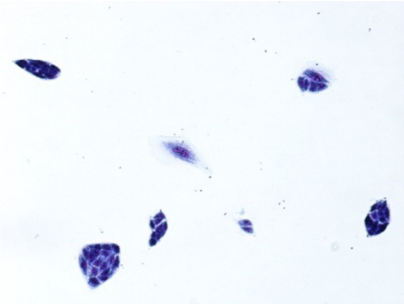

K1K1S2 30 pM

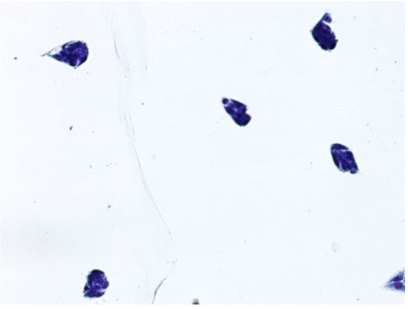

K1K1S2 10 pM

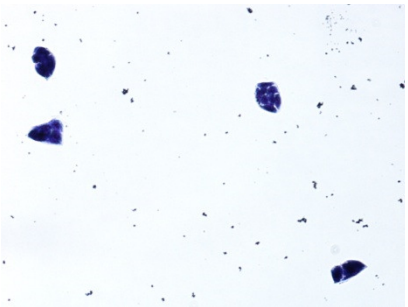

40X Nikon Eclypse bright field

Images Data Source File

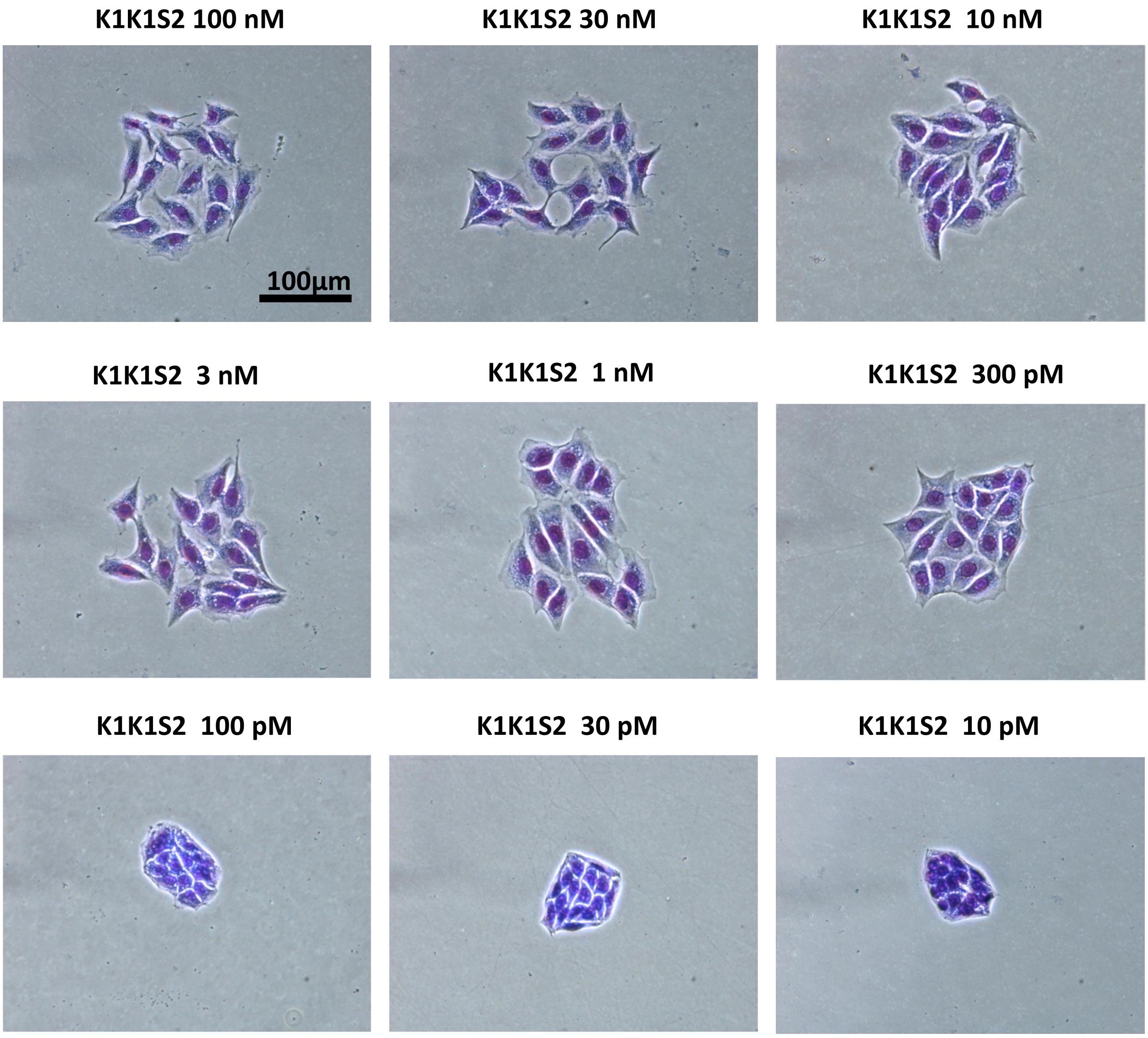

200X Nikon Eclypse bright field

Images Data Source File

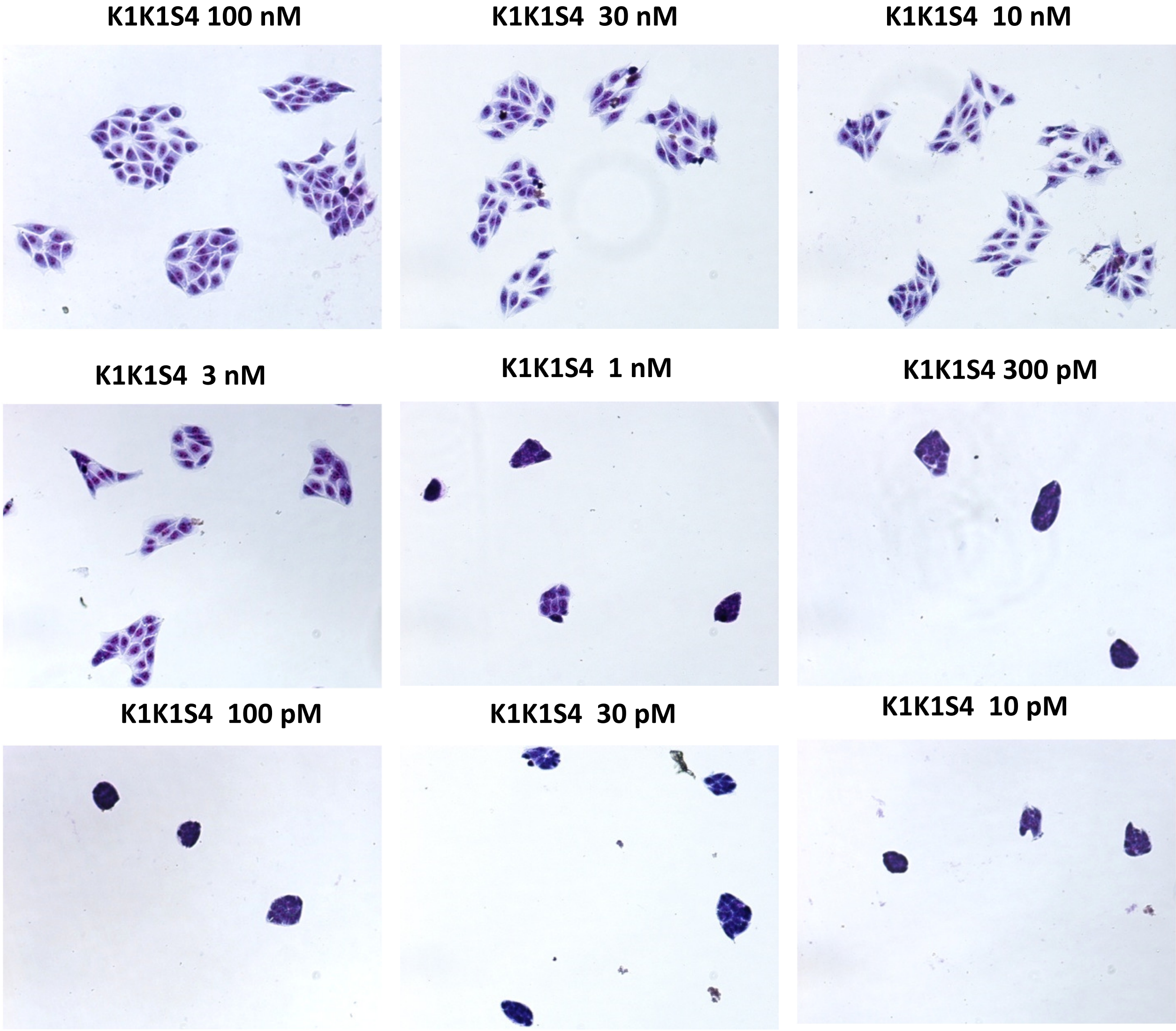

40X Nikon Eclypse bright field

Images Data Source File

K1K1S4 100 nM

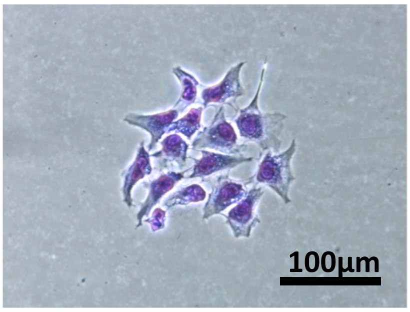

K1K1S4 30 nM

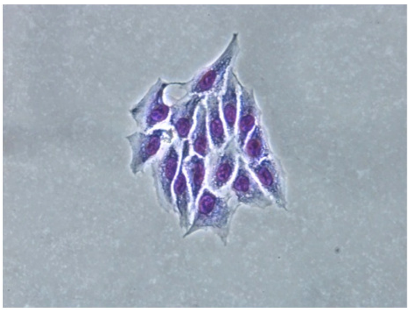

K1K1S4 10 nM

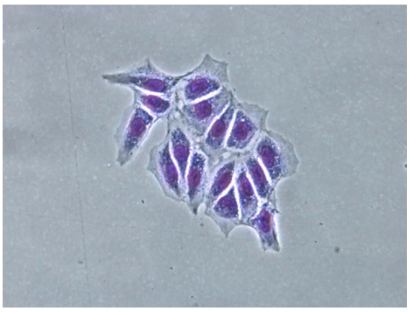

K1K1S4 3 nM

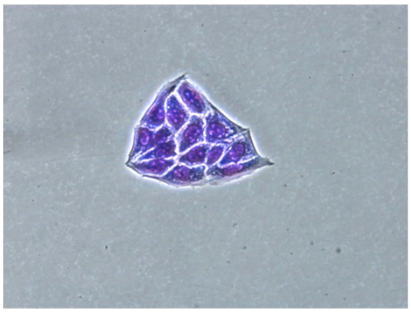

K1K1S4 1 nM

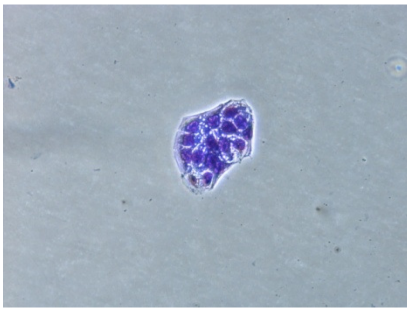

K1K1S4 300 pM

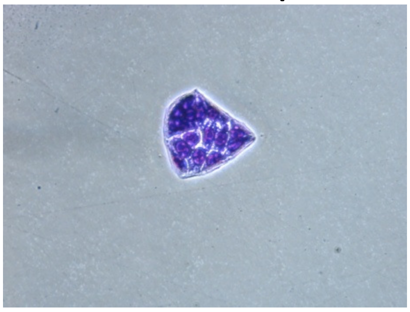

K1K1S4 100 pM

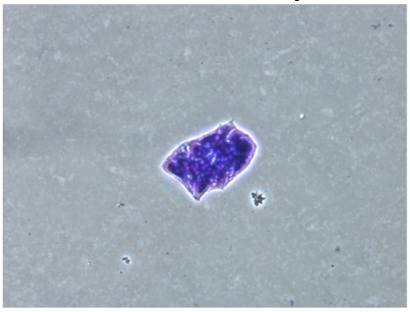

K1K1S4 30 pM

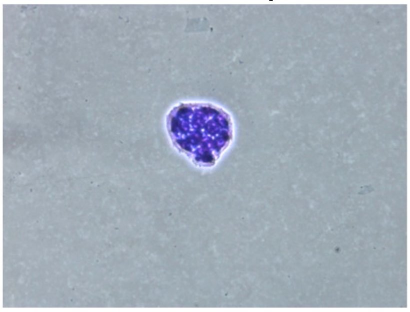

K1K1S4 10 pM

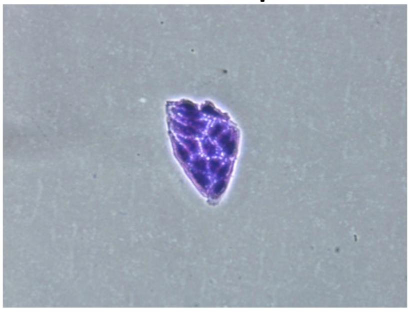

200X Nikon Eclypse Bright Field
