# Supplementary material for: Dimerization of kringle 1 domain from hepatocyte growth factor/scatter factor provides a potent MET receptor agonist
Source: Life Sci Alliance. 2022 Jul 29;5(12):e202201424. doi: 10.26508/lsa.202201424 (PMC9348577; doi:10.26508/lsa.202201424)
Supplement: Supplementary file 13 [file LSA-2022-01424_TableS1.docx]

**Supplementary Tables:**

**Table S1. Summary of X-ray data collection and refinement statistics.**

|  | **K1K1H6** | **K1K1** |
| --- | --- | --- |
| **Data Collection** |  |  |
| BeamLine | ESRF ID23-1 | ESRF BM14 |
| Wavelength | 0.9763 | 0.953725 |
| Resolution range (Å) | 35.89 - 1.7 (1.761 - 1.7) | 43.29 - 1.8  (1.864 - 1.8) |
| Space group | P1 21 1 | P1 21 1 |
| Unit cell a, b, c (Å)  α, β, γ (°) | 31.8, 59.6, 46.3 90, 103.8, 90 | 45.57, 58.75, 65.94  90, 108.219, 90 |
| Total reflections | 69988 (6427) | 161096 (16039) |
| Unique reflections | 18549 (1864) | 30781 (3066) |
| Completeness (%) | 99.81 (99.84) | 99.66 (99.64) |
| Multiplicity | 3.8 (3.4) | 5.2 (5.2) |
| CC_1/2_ | 0.999 (0.971) | 0.986 (0.824) |
| **Refinement** |  |  |
| R_work_ (R_free_) | 0.1651 (0.1942) | 0.1966 (0.2268) |
| No. of non-hydrogen atoms | 1572 | 2912 |
| Wilson B-factor | 15.16 | 20.27 |
| Average B-factor  RMS (bonds)  RMS (angles)  Ramachandran favored (%)  Ramachandran allowed (%)  Ramachandran outliers (%) | 22.03  0.020  1.68  97.09  2.91  0.00 | 30.04  0.003  0.62  95.78  4.22  0.00 |

*N.B. Statistics for the highest-resolution shell are shown in parentheses.*
