# Supplementary material for: Dimerization of kringle 1 domain from hepatocyte growth factor/scatter factor provides a potent MET receptor agonist
Source: Life Sci Alliance. 2022 Jul 29;5(12):e202201424. doi: 10.26508/lsa.202201424 (PMC9348577; doi:10.26508/lsa.202201424)
Supplement: Supplementary file 14 [file LSA-2022-01424_TableS2.docx]

**Supplementary Tables:**

**Table S2. Summary of SAXS data analysis.**

|  | **K1K1** | **MET567** | **K1K1+MET567** |
| --- | --- | --- | --- |
| **Data Collection** |  |  |  |
| BeamLine | ESRF BM29 | ESRF BM29 | ESRF BM29 |
| Beam energy (keV) | 12.5 | 12.5 | 12.5 |
| Sample-detector distance (m) | 2.867 | 2.867 | 2.867 |
| Exposure time (s) | 1 | 1 | 1 |
| Sample cell thickness (mm) | 1 | 1 | 1 |
| Sample concentration (mg/mL) | 8.5 mg/mL | 7.6 mg/mL | 12.4 mg/mL |
| Temperature (°C) | 20 | 20 | 20 |
| Final q range (nm^-1^) | 0.01 - 4 | 0.01 - 4 | 0.01 - 4 |
| **Data Analysis** |  |  |  |
| Points used for Guinier analysis | 1-94 | 11-48 | 2-28 |
| Guinier qR_g_ limits | 1.30 | 0.97 | 0.99 |
| Guinier R_g_ (nm) | 2.22 | 3.23 | 3.78 |
| I(0) (mm^-1^) | 14.8 ± 0.01 | 64.9 ± 0.04 | 69.9 ± 0.08 |
| D_max_ (nm) | 6.6 | 11.5 | 14.2 |
| MW estimation (V_c_ based) (kDa) | 16.5 | 66.2 | 83.4 |
